# Differential Association of 4E-BP2-Interacting Proteins Is Related to Selective Delayed Neuronal Death after Ischemia

**DOI:** 10.3390/ijms221910327

**Published:** 2021-09-25

**Authors:** Emma Martínez-Alonso, Natalia Guerra-Pérez, Alejandro Escobar-Peso, Ignacio Regidor, Jaime Masjuan, Alberto Alcázar

**Affiliations:** 1 Department of Research, Hospital Universitario Ramón y Cajal, IRYCIS, Ctra. Colmenar km 9.1, 28034 Madrid, Spain; emma.martinez@hrc.es (E.M.-A.); natalgue@ucm.es (N.G.-P.); alejandro.escobar@hrc.es (A.E.-P.); 2 Proteomics Unit, Hospital Universitario Ramón y Cajal, IRYCIS, Ctra. Colmenar km 9.1, 28034 Madrid, Spain; 3 Department of Genetics, Physiology and Microbiology, Faculty of Biological Sciences, Complutense University of Madrid, Av. Complutense, 28040 Madrid, Spain; 4 Department of Neurophysiology, Hospital Universitario Ramón y Cajal, IRYCIS, Ctra. Colmenar km 9.1, 28034 Madrid, Spain; ignacio.regidor@salud.madrid.org; 5 Department of Neurology, Hospital Universitario Ramón y Cajal, IRYCIS, Ctra. Colmenar km 9.1, 28034 Madrid, Spain; jaime.masjuan@salud.madrid.org; 6 Department of Neurology, Facultad de Medicina, Universidad de Alcalá, Ctra. Madrid-Barcelona km 33.6, 28871 Alcalá de Henares, Spain

**Keywords:** cerebral ischemia, eIF4E-binding protein, eIF4E, protein synthesis regulation, protein complexes, neuronal death, vulnerable regions, neuroprotection, proteomics

## Abstract

Cerebral ischemia induces an inhibition of protein synthesis and causes cell death and neuronal deficits. These deleterious effects do not occur in resilient areas of the brain, where protein synthesis is restored. In cellular stress conditions, as brain ischemia, translational repressors named eukaryotic initiation factor (eIF) 4E-binding proteins (4E-BPs) specifically bind to eIF4E and are critical in the translational control. We previously described that 4E-BP2 protein, highly expressed in brain, can be a molecular target for the control of cell death or survival in the reperfusion after ischemia in an animal model of transient cerebral ischemia. Since these previous studies showed that phosphorylation would not be the regulation that controls the binding of 4E-BP2 to eIF4E under ischemic stress, we decided to investigate the differential detection of 4E-BP2-interacting proteins in two brain regions with different vulnerability to ischemia-reperfusion (IR) in this animal model, to discover new potential 4E-BP2 modulators and biomarkers of cerebral ischemia. For this purpose, 4E-BP2 immunoprecipitates from the resistant cortical region and the vulnerable hippocampal *cornu ammonis* 1 (CA1) region were analyzed by two-dimensional (2-D) fluorescence difference in gel electrophoresis (DIGE), and after a biological variation analysis, 4E-BP2-interacting proteins were identified by matrix-assisted laser desorption/ionization-time of flight (MALDI-TOF) mass spectrometry. Interestingly, among the 4E-BP2-interacting proteins identified, heat shock 70 kDa protein-8 (HSC70), dihydropyrimidinase-related protein-2 (DRP2), enolase-1, ubiquitin carboxyl-terminal hydrolase isozyme-L1 (UCHL1), adenylate kinase isoenzyme-1 (ADK1), nucleoside diphosphate kinase-A (NDKA), and Rho GDP-dissociation inhibitor-1 (Rho-GDI), were of notable interest, showing significant differences in their association with 4E-BP2 between resistant and vulnerable regions to ischemic stress. Our data contributes to the first characterization of the 4E-BP2 interactome, increasing the knowledge in the molecular basis of the protection and vulnerability of the ischemic regions and opens the way to detect new biomarkers and therapeutic targets for diagnosis and treatment of cerebral ischemia.

## 1. Introduction

Eukaryotic initiation factor (eIF) 4E-binding protein 2 (4E-BP2) belongs to a group of 4E-binding translational repressor proteins (4E-BP1, 4E-BP2, and 4E-BP3), which binds specifically to eIF4E and inhibit its function. Crystal structure analysis revealed a higher binding selectivity of eIF4E to 4E-BP2 than to 4E-BP1 [1]. Active (hypo- or de-phosphorylated) forms of 4E-BPs translational repressors compete with eIF4G factor in their union with eIF4E, avoiding eIF4F complex formation and inhibiting cap-dependent translation [2,3]. It is well-known that 4E-BP1 is a substrate of mammalian target of rapamycin (mTOR), a serine/threonine-protein kinase in the phosphoinositide 3-kinase (PI3K)/protein kinase B (PKB or Akt)/mTOR signaling pathway, which is the main catalytic component of mTOR complex 1 (mTORC1) [4,5]. In response to extracellular stimuli such as energy, oxidative stress and low nutrient availability [6], mTOR phosphorylates 4E-BP1, releasing eIF4E from 4E-BP1 binding and leading to protein synthesis activation [7,8]. Conversely, a reduction in energy status—as the one caused by hypoxia—significantly decreases mTOR activity [9], which in turn results in (hypo)dephosphorylation of 4E-BP1 and inducing translation inhibition [10,11]. 4E-BP1 and 4E-BP2 are detected in many tissues, being 4E-BP2 mostly expressed in brain [12]. However, while translation regulation by 4E-BP1 phosphorylation has been deeply characterized [7,8,10,11], 4E-BP2 regulation mechanism is still unclear, and other mechanisms different to phosphorylation are involved, despite 4E-BP2 shares with 4E-BP1 the phosphorylation regulation sites, [13,14].

Brain ischemia, caused by a cerebral blood flow decrease, produces a transient period of hypoxia that results in a reduction of energy stores levels [15,16]. Both cerebral blood flow and energy production are restored in a following reperfusion period, although in early post-reperfusion stages, reactive oxygen species and an additional cellular stress are generated [17]. During ischemia-reperfusion (IR), protein synthesis remains strongly inhibited and its recovery time, much slower than energy metabolism, is dependent on the intensity and duration of the ischemic process [18,19]. Brain regions where protein synthesis is not restored suffer neuronal degeneration induced by IR stress, known as delayed neuronal death [19,20]. Hippocampal *cornu ammonis* 1 (CA1) are particularly ischemia-susceptible areas to global cerebral ischemia, being considered as a vulnerable region in contrast to the resistant regions of the cerebral cortex. IR mechanism of protein synthesis inhibition is not well established. Different types of signals in which IR stress is involved induce changes in initiation factors activity (such as eIF4E), being often these changes by phosphorylation and leading to translation inhibition [17,21]. In this sense, previous studies in our group revealed that specific changes in the binding of 4E-BP2 to eIF4E during IR could be a critical control point for protein synthesis regulation and cell decision for protection or vulnerability under ischemic stress [22]. Moreover, we demonstrated that 4E-BP2 phosphorylation is not the unique regulation for the binding of 4E-BP2 to eIF4E in IR stress [14]. In fact, differences in 4E-BP2/eIF4E association were observed after IR stress without changes in 4E-BP2 phosphorylation or other post-translational modification between the cerebral cortex and CA1 region, with opposite consequences on protein synthesis recovery [14].

With this knowledge, we considered of particular interest to elucidate if other 4E-BP2-interacting proteins different to eIF4E could be related to the changes observed in 4E-BP2/eIF4E association after IR stress. For this purpose, here we study for the first time the interactome of 4E-BP2 and identify new 4E-BP2-interacting proteins in two brain regions with different vulnerability after ischemic injury. These interacting proteins were differentially detected and related to the specific susceptibility of the brain region after IR. To investigate the interactome of 4E-BP2, we have carried out 4E-BP2 immunoprecipitations with specific anti-4E-BP2 antibodies in cortical and CA1 region tissue extracts from ischemic animals. The 4E-BP2 immunoprecipitates were analyzed by two-dimensional (2-D) fluorescence difference in gel electrophoresis (DIGE) and matrix-assisted laser desorption/ionization-time of flight (MALDI-TOF) mass spectrometry (MS). Our results report several 4E-BP2-interacting proteins, and interestingly some of them showed significant changes in their association with 4E-BP2 between resistant and vulnerable brain regions after IR, which we suggest as potential biomarkers of protection and vulnerability under ischemic stress.

## 2. Results

### 2.1. Differential Detection of eIF4E-Binding Protein 2 (4E-BP2)-Interacting Proteins in Response to Ischemia-Reperfusion (IR)

In our study we used the four-vessel occlusion model to induce transient global cerebral ischemia. We decided to examine the effect after 3 days of ischemic reperfusion (R3d), considering that a significant neuronal death at hippocampal CA1 region was observed at this time point, as we previously reported [22]. To evaluate the differential detection of 4E-BP2-interacting proteins and its potential link to a different susceptibly to brain damage, we studied the cerebral cortex and hippocampal CA1 region as the main resistant and vulnerable regions, respectively, to ischemia-reperfusion damage [20,23].

Several anti-4E-BP2 antibodies from different commercial sources were tested for immunoprecipitation by Western blot. To assess the specificity, 4E-BP2 immunoprecipitates were analyzed by Western blot with anti-4E-BP1 antibody (Supplementary Figure S1). The results showed that 4E-BP1 was not detected in the immunoprecipitates, but it remained mainly in the non-precipitated fraction, along with almost undetectable amounts of 4E-BP2 (Figure S1). Anti-4E-BP2 supplied by Sigma-Aldrich (E6532; Merck KGaA, Darmstadt, Germany) was identified as the most specific and effective for the obtaining of 4E-BP2 immunoprecipitates, and was the used in this study (Figure S1).

**Figure 1:**
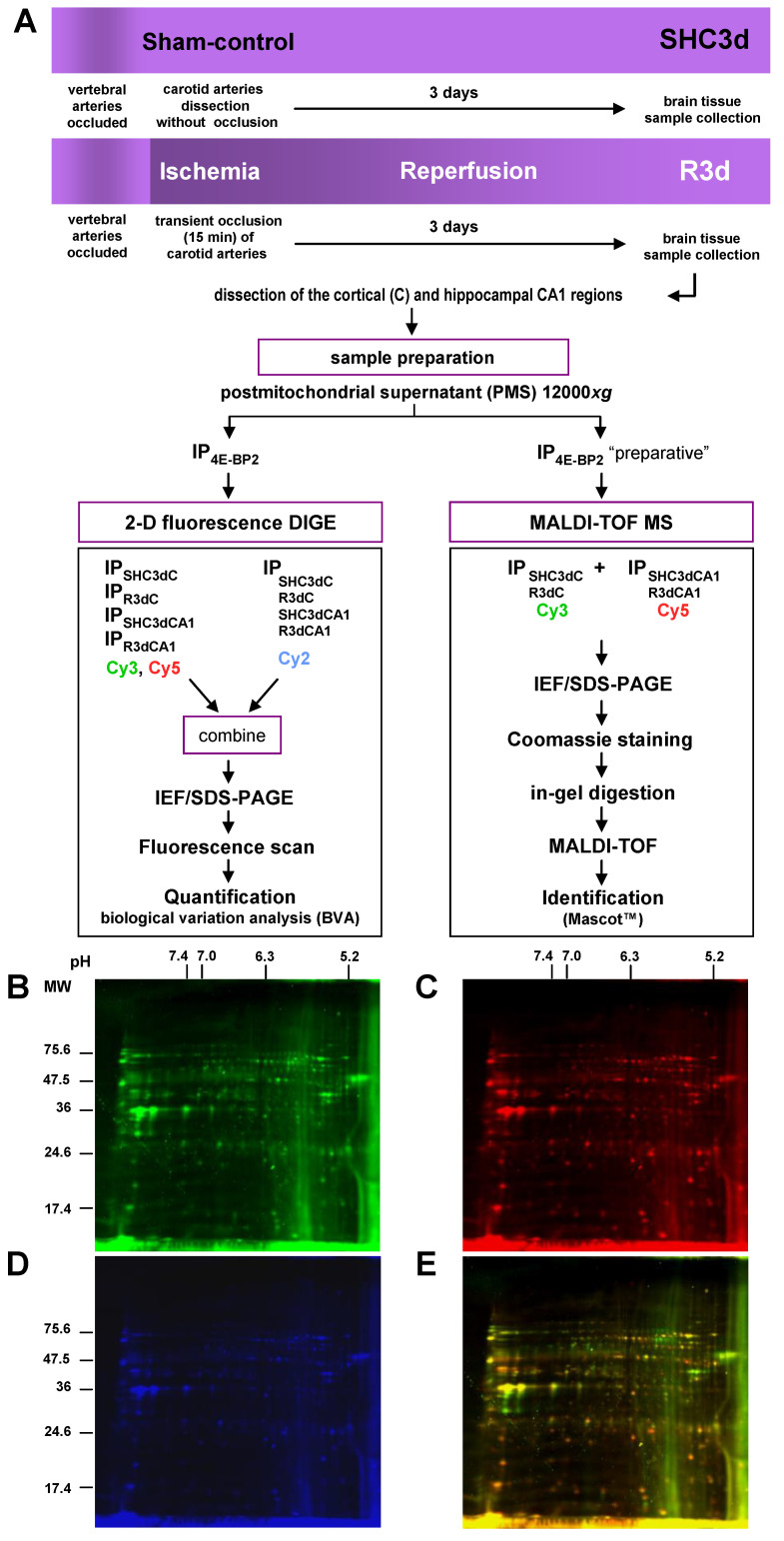
Identification strategy of eIF4E-binding protein 2 (4E-BP2)-interacting proteins in response to ischemia-reperfusion (IR). (**A**) Flow chart of the experimental animal model of ischemia-reperfusion, sample preparation, and quantification and identification strategy. (**B**–**E**) Representative scanned images of two-dimensional (2-D) fluorescence difference in gel electrophoresis (DIGE) of 4E-BP2 immunoprecipitates from ischemic animals combining immunoprecipitates of the cerebral cortex and CA1 region, labelled with Cy3 (in green, **B**) and Cy5 (in red, **C**), respectively, and the internal standard pool labelled with Cy2 (in blue, **D**). The overlay of Cy3 and Cy5 fluorescence scanned images is shown in (**E**). Abbreviations: SHC3dC and SHC3dCA1, samples of the cerebral cortex (C) and hippocampal *cornu ammonis* 1 (CA1) region, respectively, from sham control animals; R3dC and R3dCA1, samples of the cerebral cortex and CA1 region, from ischemic animals after 3 days of reperfusion.; IP, immunoprecipitates of 4E-BP2; IEF, isoelectric focusing; SDS-PAGE, sodium dodecyl sulphate-polyacrylamide gel electrophoresis. Fluorescence gel images (**B**–**E**) are full original images; the horizontal axis represents pH and the vertical axis represents molecular weight (MW, in kDa) from protein markers.

**Figure 2:**
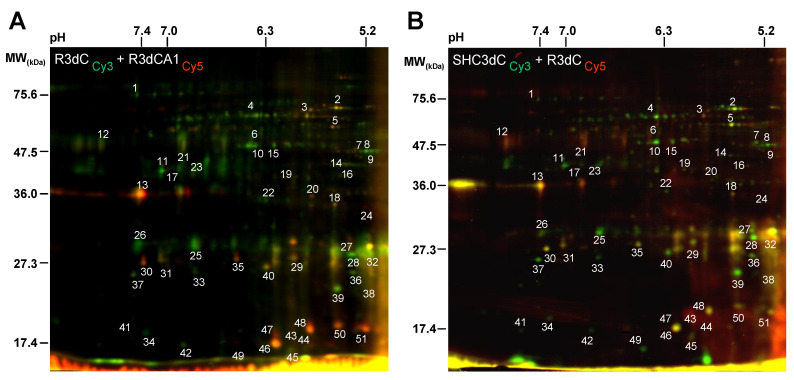
Differential detection of 4E-BP2-interacting proteins in response to ischemia-reperfusion. (**A**) 2-D DIGE scanned images overlay of the 4E-BP2 immunoprecipitate from R3dC sample, labelled with Cy3 (green), combined with the 4E-BP2 immunoprecipitate from R3dCA1, labelled with Cy5 (red), comparing cerebral cortex and CA1 samples from ischemic animals (R3dC/R3dCA1 combination). (**B**) 2-D DIGE scanned images overlay of the 4E-BP2 immunoprecipitate from SHC3dC control sample, labelled with Cy3 (green), combined with the 4E-BP2 immunoprecipitate from R3dC, labelled with Cy5 (red), comparing cerebral cortex samples from control and ischemic animals (SHC3dC/R3dC combination). Yellow color is due to the merge of green and red fluorescence labels. The images are representative of eight different experiments performed in paired combination of four different samples (*n* = 4) from each of the experimental groups (SHC3dC, SHC3dCA1, R3dC and R3dCA1), and each one of them from a pool of four independent animals. Gel images are full original images; the horizontal axis represents pH and the vertical axis represents molecular weight (MW, in kDa). Detected proteins (spots) in all samples were quantified and spots that showed a differential intensity were tagged with numbers.

Next, we designed an experimental approach to identify 4E-BP2-interacting proteins in those two cerebral regions with different response to IR. 4E-BP2 immunoprecipitated samples of cerebral cortex and CA1 region from both controls and ischemic animals were labelled with fluorescent Cy3 and Cy5 dyes for 2-D DIGE analysis. Since the protein content in 4E-BP2 immunoprecipitates was very low, PMS samples from four different animals of each experimental condition were pooled in order to obtain enough amount of protein in the 4E-BP2 immunoprecipitates for the 2-D DIGE procedure. Then, four independent 4E-BP2 immunoprecipitates (*n* = 4 sample sets, from 16 animals) of cortical and CA1 samples from control animals (SHC3dC and SHC3dCA1 samples, respectively), and other four 4E-BP2 immunoprecipitated samples of these studied brain regions from ischemic animals (R3dC and R3dCA1 samples, respectively) were analyzed by 2-D DIGE. Before 2-D DIGE, each sample set was labelled on cysteines with Cy3 or Cy5 dyes and labelled 4E-BP2 immunoprecipitates of each of the different experimental groups (*n* = 4) were combined in eight paired computer assisted combinations and mixed with the internal standard pool (Figure 1). The eight different pairs of combinations between the different experimental groups were R3dC_1_/R3dCA1_1_, R3dC_3_/R3dCA1_2_, SHC3dC_1_/R3dC_2_, R3dCA1_4_/SHC3dCA1_3_, SHC3dC_4/_SHC3d3dCA1_4_, R3dCA1_3_/SHC3dC_2_, SHC3dCA1_1_/SHC3dC_3_ and SHC3dCA1_2_/R3dC_4_, labeled with Cy3/Cy5, respectively (the number in subscript was each of the sample sets analyzed), and compared all experimental conditions with each other and resolved by 2-D DIGE. Afterwards, the 4E-BP2 interactome was visualized using a fluorescence scanner (Figure 1B–E). The 2-D DIGE results showed a differential association of 4E-BP2-interacting proteins (spots) between samples of the cortical resistant and CA1 vulnerable regions, whose protein levels were directly proportional to the labelled protein intensity (Figure 2A). Spots with similar intensities were also found in the paired combinations of 4E-BP2 immunoprecipitates, corresponding to similar levels of 4E-BP2-associated proteins between compared experimental groups (Figure 2). For a better report and comprehension of the biological variation analysis of 4E-BP2-interaction proteome profile, representative combinations of different experimental groups are shown in Figure 2.

### 2.2. Biological Variation Analysis of the Differentially Detected 4E-BP2-Interacting Proteins

**Figure 3:**
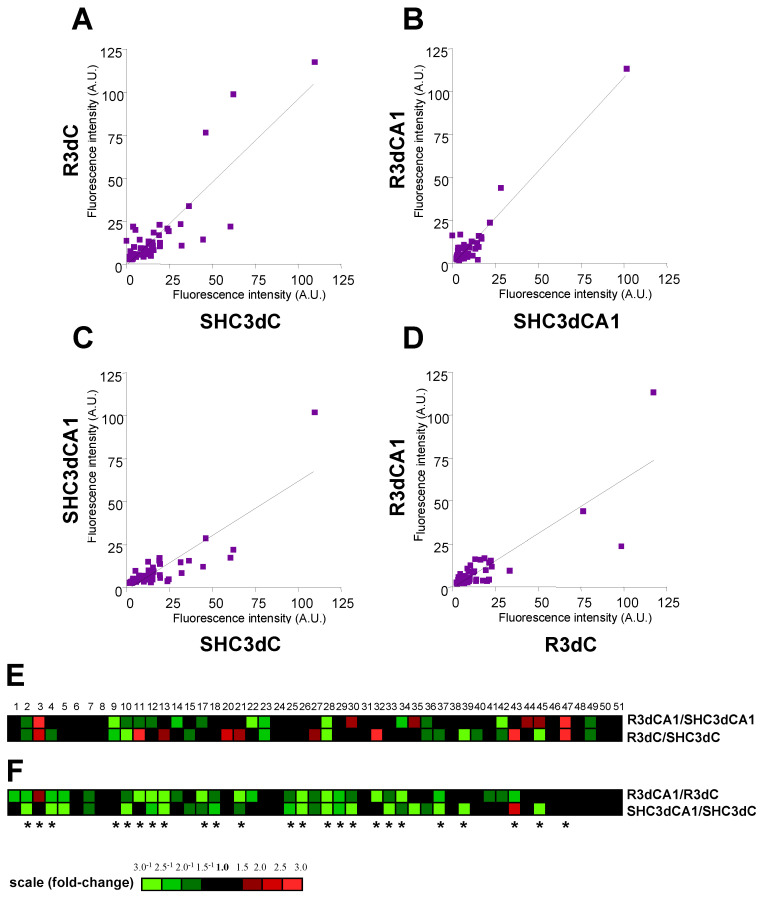
Different brain regions showed a differential association of 4E-BP2-interacting proteins. Scatter plots of the fluorescence intensity of detected proteins in 4E-BP2 immunoprecipitates in ischemia-reperfusion were represented against control condition, in the cerebral cortex (R3dC vs. SHC3dC) (**A**) and CA1 region (R3dCA1 vs. SHC3dCA1) (**B**). Scatter plots of the fluorescence intensity of detected proteins in the CA1 region were also represented against the cerebral cortex in control and IR condition (SHC3dCA1 vs. SHC3dC, and R3dCA1 vs. R3dC) (**C**,**D**). Fluorescence intensity was quantified in arbitrary units (A.U.). One dot represents the mean of four experiments. The linear regression fitting line, and Spearman correlation *p* values were < 0.0001. Heat maps of 49 proteins associated to 4E-BP2 were obtained as fold-changes in IR with respect to their control in both CA1 (ratio R3dCA1/SHC3dCA1) and cortical (ratio R3dC/SHC3dC) regions (**E**), and in the CA1 region with respect to the cerebral cortex in IR (ratio R3dCA1/R3dC) and control (ratio SHC3dCA1/SHC3dC) conditions (**F**). Found 4E-BP2-associated proteins were tagged with numbers, and color scale illustrates their fold-change. Differentially associated proteins among the four experimental groups, SHC3dC, SHC3dCA1, R3dC, and R3dCA1, were assessed by ANOVA test (* *p* < 0.05).

To assess differences in protein association with 4E-BP2, 4E-BP2-interacting proteins were quantified for biological variation analysis (BVA) in scanned DIGE gels. Quantification data for the protein spots found in the IR condition were compared to those same spots in the control condition (i.e., R3dC vs. SHC3dC, and R3dCA1 vs. SHC3dCA1), and those of the CA1 region were compared to the ones in the cortical region (i.e., SHC3dCA1 vs. SHC3dC, and R3dCA1 vs. R3dC). Fluorescence intensity of each protein was plotted on scatter diagrams for each comparison, representing in the *x* and *y* axes the aforementioned experimental conditions (Figure 3A–D). These comparisons showed a linear relationship for most of the proteins analyzed, being also significantly correlated (*p* < 0.0001, by Spearman’s test) in all cases (Figure 3A–D). In particular for the analysis of IR compared with control conditions (Figure 3A,B), a higher number of proteins with different fluorescence and therefore different association levels —i.e., outliers— can be observed in the cerebral cortex than in the hippocampal CA1 region, meaning a higher influence of the ischemic stress on the association of proteins within the cortex. When both regions were compared (Figure 3C,D), the slope of the regression line indicated higher levels of 4E-BP2 proteins in the cerebral cortex, both for control and ischemic conditions.

In order to analyze which particular proteins showed differences in response to IR stress, either in cortical or in CA1 regions, we performed a clustering analysis of the quantified protein spots. Figure 3E represents the heat map obtained from intensity fluorescence ratio (fold-change) in IR with respect to their control condition in both cortical and CA1 regions (Figure 3E, R3dC/SHC3dC and R3dCA1/SHC3dCA1 ratios, respectively). The BVA revealed quantitative changes in 51 proteins, although two spots (16 and 19) were excluded as labelling artefacts. Among these differentially detected proteins, the BVA revealed six 4E-BP2-interacting proteins in the CA1 region (vulnerable region) (proteins 3, 30, 35, 44, 45, and 47), and nine proteins in the cerebral cortex (resistant region) (proteins 3, 11, 13, 20, 21, 27, 32, 43, and 47) with increased levels (≥1.5-fold) induced by IR stress (Figure 3E). Conversely, fourteen 4E-BP2-interacting proteins had decreased levels (≥1.5-fold) in both cortical and CA1 regions under IR stress (Figure 3E).

When the ratio between CA1 and cortical region was calculated in both control and IR condition (Figure 3F, SHC3dCA1/SHC3dC and R3dCA1/R3dC ratios, respectively) an increase of 4E-BP2 association (≥1.5-fold) was observed in the protein 3 in R3dCA1/R3dC, and in protein 43 in SHC3dCA1/SHC3dC. On the contrary, the cerebral cortex had increased levels (≥1.5^−1^-fold) in a total of 27 and 24 proteins, in IR and control, respectively, with respect to the CA1 region (Figure 3F). These results agree with the scatterplots described above, which showed lower levels—i.e., a lower slope—of 4E-BP2-interacting proteins in the CA1 region.

ANOVA test was performed in order to determine a significant biological variability in 4E-BP2-interacting proteins between the four experimental groups (SHC3dC, SHC3dCA1, R3dC, and R3dCA1). Test results provided 24 proteins with significant ANOVA (*p* < 0.05), proteins that also showed ≥1.5-fold change in their levels (Figure 3E,F).

### 2.3. Identification of 4E-BP2-Interacting Proteins

The biological variation analysis of 4E-BP2-interacting proteins in the cerebral cortex and CA1 region revealed significant and differential changes in response to IR stress (Figure 3). To identify these proteins, a preparative 2-D fluorescence DIGE was performed (Figure S2). The DIGE gel was scanned, then stained with Coomassie brilliant blue solution, and protein spots were matched between the fluorescence scanned image and the Coomassie stained gel (Figure S2). Among the previously detected 49 spots, six of them (proteins 21, 27, 46, 48, 50, and 51) were not found in the Coomassie stained gel. The protein spots were excised from the stained gel, in-gel digested with trypsin and identified by MALDI-TOF MS. The identification was further confirmed by MS in tandem MALDI LIFT-TOF/TOF as described in Methods section.

From the 49 proteins analyzed, 31 were identified by MALDI-TOF MS, corresponding to 30 different proteins, including 20 out of 24 proteins differentially detected by BVA with significant biological changes in response to IR stress (Figure 3F). These proteins are included in Table 1. Additionally, 11 proteins out of 25 with no significant variation were also identified (Supplementary Table S1). In these protein identifications, the corresponding apparent molecular mass and pI, sequence coverage, and score were determined. For every identification, the apparent molecular mass and pI were coherent with their theoretical values. Score was obtained from Mascot search algorithm as an identification quality indicator. Any score higher than 51 in the MALDI-TOF MS analysis, and higher than 24 in the MALDI LIFT-TOF/TOF MS/MS analysis was considered significant (*p* < 0.05); all identified proteins had significant high scores (Table 1 and Table S1).

Among the proteins analyzed by MALDI-TOF MS (Table 1 and Table S1), 4E-BP2 and eIF4E were not identified, despite 4E-BP2 being the target protein for immunoprecipitation, and eIF4E a known 4E-BP2-interacting protein [1] and therefore they are expected to be present in the immunoprecipitated samples as well. In the case of 4E-BP2, it has been described that 4E-BP2 is not identified by MS, probably due to a deficient peptide ionization [24]. To overcome this issue and detect these proteins, we resolved a 2-D sodium dodecyl sulphate-polyacrylamide gel electrophoresis (2-D SDS-PAGE) with 4E-BP2 immunoprecipitated samples of cerebral cortex (SHC3dC and R3dC) run in parallel to the DIGE experiment of Figure 2, and incubated with anti-4E-BP2 and anti-eIF4E antibodies after gel blotting (Figure S3A). 4E-BP2 was detected by anti-4E-BP2 antibody, corresponding to protein spots 46, 48, 50, and 51 by their exact matching with 2-D-DIGE gel of Figure 2 (Figure S3A,B). As expected, these protein spots did not have differences in their levels between the different conditions analyzed (Figure 3E,F). In addition, eIF4E was also identified by anti-eIF4E antibody (Figure S3A). In this case, it could not be matched to any spot on the 2-D DIGE gel (Figure 2), probably due to a lack of labelling by Cy-dyes. However, eIF4E had been detected in 4E-BP2 immunoprecipitates from all these studied samples—SHC3d control and R3d ischemic samples from both cortical and CA1 regions—by Western blotting using the same antibody as previously described by our group [14] (Figure S3C).

### 2.4. Changes in the Levels of 4E-BP2-Interacting Proteins in the Cerebral Cortex and Hippocampal CA1 Region under IR. Identification of Biomarkers of Protection or Vulnerability in Cerebral Ischemia

**Figure 4:**
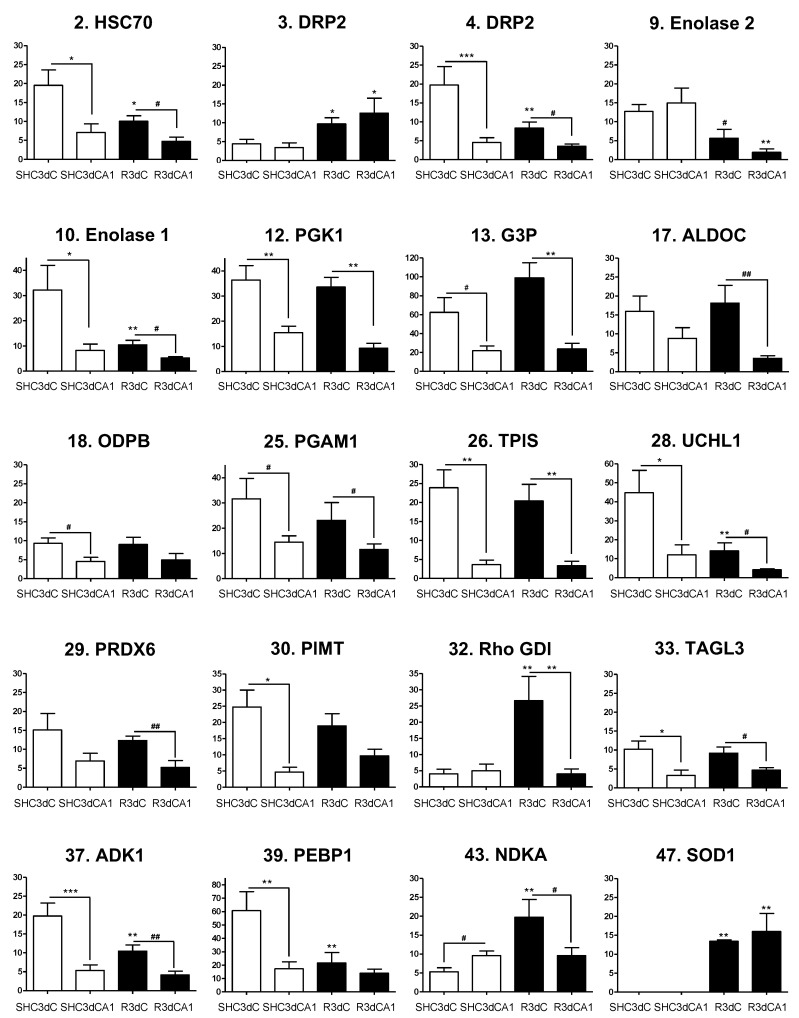
Levels and statistical analysis of identified 4E-BP2-interacting proteins with significant differential changes. Bar graphs show the 4E-BP2-associated protein levels in control (SHC3d) and ischemic samples (R3d) from the cerebral cortex (SHC3dC and R3dC) and hippocampal CA1 region (SHC3dCA1 and R3dCA1). Data are represented as mean of *n* = 4 independent samples from 2-D DIGE experiments. Error bars indicate SE. Scheme 0. ** *p* < 0.01; *** *p* < 0.001) or by Student’s t-test (# *p* < 0.05; ## *p* < 0.01), after significant ANOVA (* *p* < 0.05), compared with their respective control, or between the cerebral cortex and CA1 samples (indicated by lines). The vertical axis indicates the quantification values of the spot intensity in arbitrary units. Graphs are numbered and named according to the proteins listed in Table 1.

When proteins that interact with 4E-BP2 were identified, we explored whether these proteins could be related to IR stress, and, furthermore, to the protection or vulnerability of the studied regions in response to IR. To this end, we performed multiple statistical comparisons between resistant (cerebral cortex) and vulnerable (CA1) regions from both control and IR conditions (Figure 4, Figures S4 and S5). Thus, for each identified protein we compared its levels in both cortical and CA1 ischemic samples with respect to their control condition, (i.e., R3dC vs. SHC3dC, and R3dCA1 vs. SHC3dCA1), and in the CA1 vulnerable region with respect to the cortical resistant region, for both control and IR conditions (i.e., SHC3dCA1 vs. SHC3dC, and R3dCA1 vs. R3dC). As a result, we identified 20 4E-BP2-interacting proteins that had statistically significantly different levels between the cerebral cortex and CA1 region, or in the IR condition compared with their respective SHC3d control (Figure 4). Other 25 proteins did not have statistical differences, among which 11 were identified (Figure S4), and 14 unidentified proteins (Figure S5). Among the latter, proteins 46, 48, 50, and 51, were identified as 4E-BP2 (Figure S3), as previously mentioned, and accordingly, they had no differences. Finally, other four unidentified proteins (11, 21, 34, and 45) also showed statistical differences (Figure S5).

**Figure 5:**
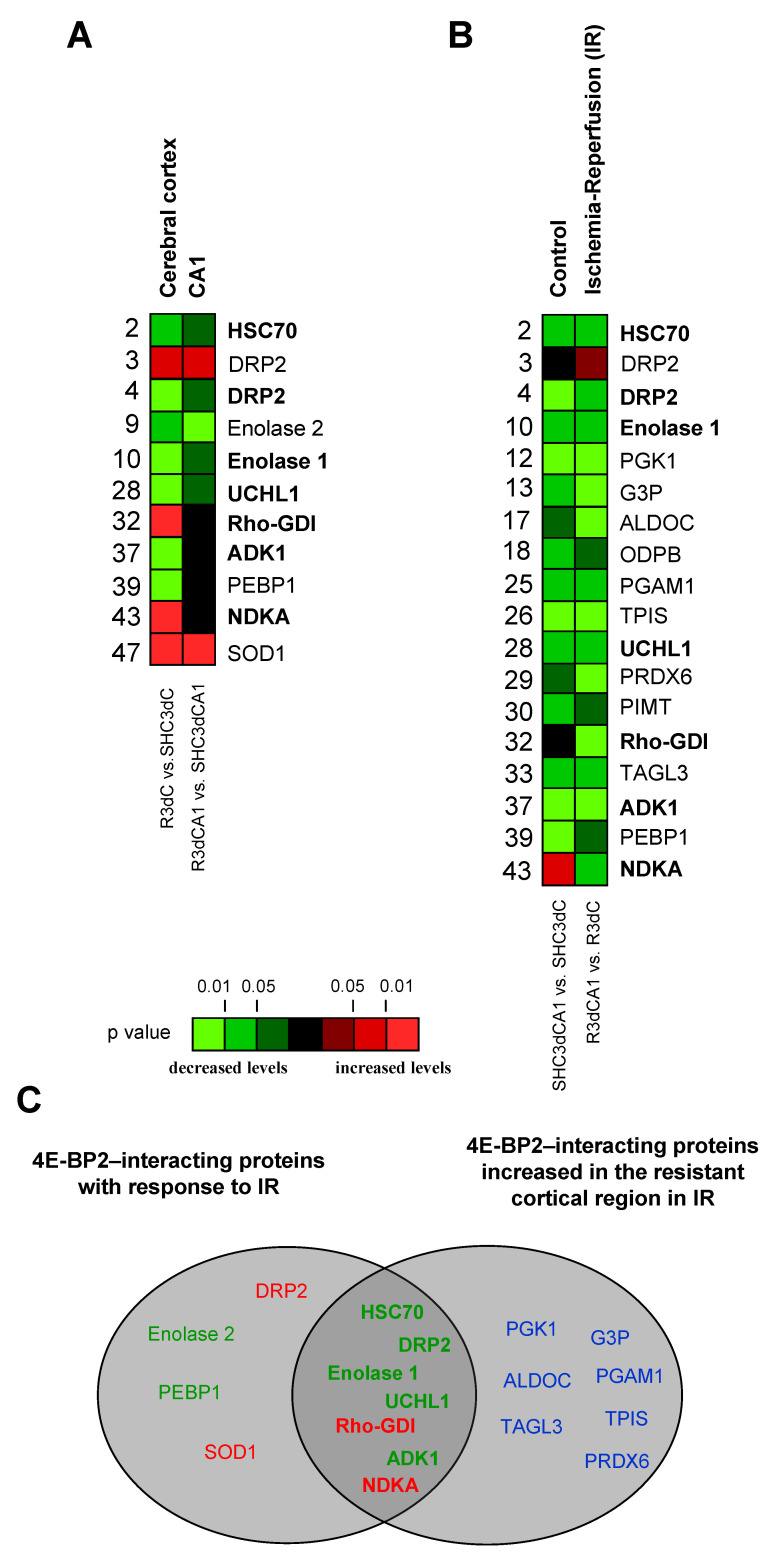
4E-BP2-interacting proteins with significant differences between the cortical and CA1 regions, in control and ischemic samples. Identified 4E-BP2-interacting proteins were quantified for biological variation analysis and data were compared between the ischemic group and their control condition for the cerebral cortex and CA1 region (R3dC vs. SHC3dC, and R3dCA1 vs. SHC3dCA1, respectively) (**A**). Other comparisons were done between the vulnerable CA1 region and the cortical resistant region, in both control and ischemia-reperfusion (IR) (SHC3dCA1 vs. SHC3dC, and R3dCA1 vs. R3dC, respectively) (**B**). Data and statistical analysis were from Figure 4, and color code represents the statistical significance (*p* value). Proteins marked in bold highlight common proteins with significant changes in R3dC and R3dCA1 ischemic samples (**A**) and between them (**B**, right column). (**C**) Schematic diagram of differentially detected 4E-BP2-interacting proteins in control and ischemic samples. The left diagram includes proteins with increased (red) or decreased (green) association with 4E-BP2 in response to IR stress, as potential ischemia biomarkers. The right diagram includes proteins with significantly lower levels of 4E-BP2 association in CA1 than in the cerebral cortex. The central intersection corresponds to common biomarkers, as potential biomarkers of protection or vulnerability in cerebral ischemia.

Thus, proteins that had significantly different levels in the cerebral cortex or CA1 region in IR (R3dC and R3dCA1, respectively) compared with their control value (SHC3d groups) could be identified as IR biomarkers (Figure 5A). In cerebral cortex we observed increased levels of dihydropyrimidinase-related protein 2 (DRP2, spot 3), Rho GDP-dissociation inhibitor 1 (Rho-GDI), nucleoside diphosphate kinase A (NDKA), and superoxide dismutase 1 (SOD1), and, in contrast, decreased levels of heat shock 70 kDa protein 8 (HSC70), DRP2 (spot 4), enolase 1 and 2, ubiquitin carboxyl-terminal hydrolase isozyme L1 (UCHL1), adenylate kinase isoenzyme 1 (ADK1), and phosphatidyl ethanolamine-binding protein 1 (PEBP1). Moreover, in CA1 region, DRP2 (spot 3) and SOD1 showed higher levels, whereas enolase 2 had significantly lower levels (Figure 5A). Furthermore, significant changes in the association levels of 4E-BP2-interacting proteins between the resistant cortical region and the vulnerable CA1 region under IR stress can be analyzed for the identification of potential biomarkers of protection or vulnerability in cerebral ischemia (Figure 5B). Eighteen proteins were identified with significant changes in control or IR condition, most of them decreased in the CA1 region, i.e., increased in the cortical region. Nevertheless, the CA1 region showed an increase in the protein levels of NDKA and DRP2 (spot 3) in the control and IR, respectively.

Taken together, eleven 4E-BP2-interacting proteins were identified with significant changes in their association in response to IR stress, and could be considered as biomarkers of cerebral ischemia (Figure 5A). Interestingly, among these, DRP2 (spot 3) and SOD1 responded to IR stress increasing their association with 4E-BP2 in both cortical and CA1 region, and Rho-GDI and NDKA specifically did so only in the cortical resistant region (Figure 5A). Furthermore, seven of them, HSC70, DRP2 (spot 4), enolase 1, UCHL1, Rho-GDI, ADK1, and NDKA, were common to those with significantly lower levels in the vulnerable CA1 region than in the cortical resistant region (Figure 5C), highlighting Rho-GDI and NDKA with a very significant increase in the cortical resistant region induced by IR. These seven proteins would be selected as biomarkers of protection or vulnerability against IR stress due to the differences in their association with 4E-BP2 (Figure 5C).

## 3. Discussion

In this study we identified the 4E-BP2 interactome in a transient global cerebral ischemia model. We describe for the first time specific 4E-BP2-interacting proteins with significant biological variation in their association with 4E-BP2, discerning protein biomarkers from resistant (cortical region) and vulnerable (hippocampal CA1) regions to ischemia-reperfusion (IR).

It is well-known that protein synthesis is inhibited during ischemia and post-ischemic reperfusion, both in global and focal cerebral ischemia [16], but it is only recovered in regions so-called resistant to ischemia-reperfusion-induced damage. Thus, there is a direct connection between translation inhibition and alterations that occur during cerebral ischemia-reperfusion, which involves ischemic tissue death [25]. In transient global cerebral ischemia, translation inhibition is reversed in most brain regions, such as cerebral cortex, and protein synthesis gradually recovered after the first few hours of reperfusion. However, in selective vulnerable regions, such as the hippocampal CA1 region, translation inhibition persists and is closely correlated with post-ischemic neuronal death [26,27,28]. To date, the causes of this persistent translation inhibition have not yet been clarified.

Taking into account the balance in favor of cell survival mechanisms in cerebral cortex, opposite to hippocampal CA1, it would be highly relevant to discriminate how the functional translation inhibition control works in ischemic reperfusion comparing these two regions with dissimilar damaging thresholds. The availability of eIF4E, component of eIF4F complex which is regulated by 4E-BPs repressor proteins, is a central mechanism in eIF4F activity and therefore in the translational control under ischemic stress. Previous studies in our laboratory showed that the regulation of eIF4F complex by the 4E-BP2/eIF4E association is a critical aspect of the persistent translation inhibition in the vulnerable CA1 region [22]; with a lower association of 4E-BP2/eIF4E in the resistant cortical region after the 3-day reperfusion period (R3d), which was independent of 4E-BP2 phosphorylation regulation [14]. An increased knowledge of proteins involved in the regulation of eIF4E and 4E-BP2 associations in resistant and vulnerable regions to ischemia-reperfusion stress would contribute to a better understanding of the neuronal death induced by ischemia-reperfusion stress, identify new targets for therapeutic intervention, and discover new potential biomarkers.

**Figure 6:**
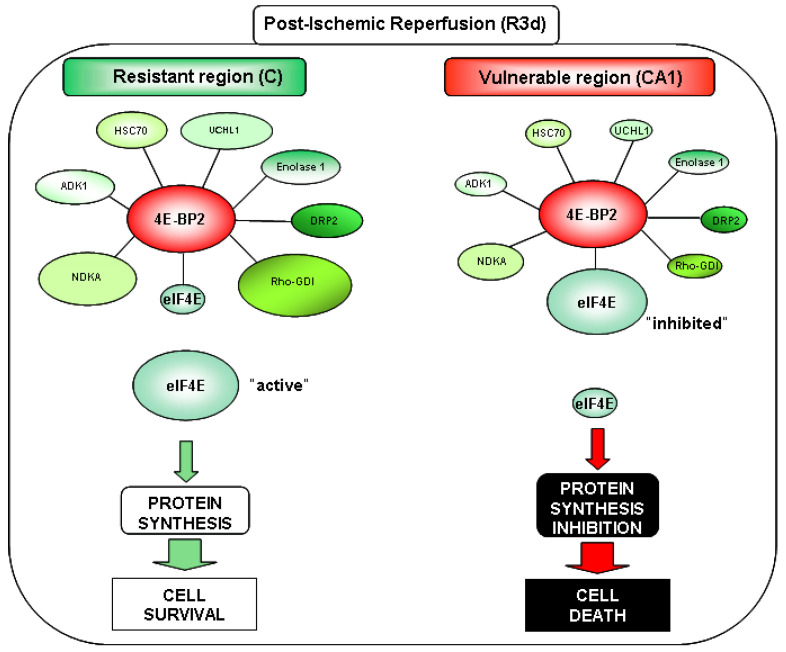
4E-BP2-interacting proteins as pivotal modulators in the cellular response to ischemia-reperfusion stress. Proposed model of 4E-BP2 regulation in resistant and vulnerable regions in brain ischemia: 4E-BP2-interacting proteins would compete with eIF4E, releasing this factor when they increase their association with 4E-BP2 (e.g., in the cerebral cortex, C), promoting protein synthesis and cell protection to ischemia-reperfusion stress. Conversely, a decreased of 4E-BP2-interacting proteins, would allow a higher eIF4E/4E-BP2 association with the subsequent protein synthesis inhibition and cell death.

The use in this report of 4E-BP2 immunoprecipitates, proteomic identification, and DIGE technology has led to the discovery of 4E-BP2-interacting proteins. The results showed that 4E-BP2 had a broad spectrum of interacting proteins. First, we found 51 proteins that could participate in the 4E-BP2 interactome in brain samples from ischemic animals. In this regard, 31 proteins were identified, from which 20 of them showed significant differences between the control and ischemia-reperfusion conditions studied. Among these 20 identified proteins, seven (HSC70, DRP2 spot 4, enolase 1, UCHL1, Rho-GDI, ADK1 and NDKA) emerged with significant changes in their association with 4E-BP2 between resistant and vulnerable regions after ischemia-reperfusion (Figure 5 and Figure 6). These proteins had higher levels in the resistant cortical region than in the vulnerable CA1 region, although some of them showed reduced levels following reperfusion. As described above, cerebral ischemia induces a protein synthesis inhibition, which is transient in resistant regions. After this transient translation inhibition, it is very feasible that some proteins do not recover their initial levels. This result would be the one observed in the proteins HSC70, DRP2 (spot 4), enolase 1, UCHL1, and ADK1 in the cerebral cortex. However, these levels were significantly higher than in the CA1 region, and possibly enough to make a difference in the cell response to ischemia-reperfusion stress. We hypothesized that proteins displaying a higher association with 4E-BP2 in the cerebral cortex could promote a 4E-BP2 capture and a release of eIF4E and the recovery of protein synthesis in this region (Figure 6), rendering it “resistant”. Conversely, these 4E-BP2-interacting protein levels are decreased in the vulnerable CA1 region, allowing a higher eIF4E/4E-BP2 association, and the subsequent protein synthesis inhibition, which finally leads to neuronal death (Figure 6). Other identified proteins of interest had decreased levels in the CA1 region (PGK1, G3P, ALDOC, PGAM1, TPIS, PRDX6, and TAGL3) (Figure 5). However, these proteins showed a similar pattern —significant higher levels in the cerebral cortex compared with CA1 region— in both control and ischemic condition. From this, it could be concluded that their differences may be due to constitutive differences between these brain regions, and not to a specific ischemia-reperfusion effect.

Although eIF4E could not be detected in 2-D DIGE experiments, possibly due to a lack of labelling by Cy-dyes, the higher eIF4E association with 4E-BP2 in the CA1 region compared with cerebral cortex in post-ischemic reperfusion (R3d) has been previously reported [14]. In addition to this issue, we are aware of two other challenges of this study: (i) the non-identification of proteins from the database; and (ii) the fact that the identified proteins might form complexes with each other, and not necessarily bind with a direct interaction between them. Despite these limitations, we have managed in this work to shed light into the complexity of the 4E-BP2 interactome with the identification of many and varied actors playing a role in the translation control during IR damage and recovery.

We hypothesized that the new seven 4E-BP2-interacting proteins shown in Figure 6 could be potential translation modulators promoting eIF4E release by a 4E-BP2-complex-mediated mechanism in response to ischemia-reperfusion stress, being translation activators that induce the recovery of protein synthesis when their association with 4E-BP2 is increased. Proteins identified with these characteristics were HSC70, DRP2, enolase 1, UCHL1, Rho-GDI, ADK1, and NDKA. Interestingly, some of these proteins have already been described in cerebral ischemia studies [29,30,31,32,33,34,35], including human patient samples.

In this study, HSC70 is found in the 4E-BP2 interactome, showing a higher association in the cerebral cortex compared with the CA1 region. HSC70 is a member of Hsp70 family, a chaperone that facilitates a proper folding of newly translated and misfolded proteins, and stabilizes or degrades mutant proteins [36]. Related to many biological processes including signal transduction, apoptosis, autophagy, protein homeostasis, cell growth and differentiation [37,38], HSC70 has been also associated with a large number of cancers, neurodegenerative diseases, cellular senescence, and aging [36]. Precisely, it is highly relevant that HSC70 has a constitutive expression in brain after focal cerebral ischemia [30,39,40,41]. Previous studies of our group identified an increase in HSC70 association with protein phosphatase 1 (PP1) in the cerebral cortex at short term reperfusion (15–30 min) after ischemia, reverting to control condition levels after 4 h of post-ischemic reperfusion [29]. All the reports described above together with our results would encourage a contribution of HSC70 in the recovery of the cortical region against a stress, e.g., cerebral ischemia.

Enolase 1 protein is a multifunctional enzyme that participates in glycolysis, growth control, hypoxia tolerance, and allergic response. Moreover, it is a plasminogen activator and receptor on cell surface of leukocytes and neurons [42]. Some authors report an increase of enolase 1 in old mice brains susceptible to oxidative stress and brain dysfunction [43]. Here, we report a higher association with 4E-BP2 in the cerebral cortex compared with the CA1 region.

UCHL1 is a highly abundant protein in the brain [44], member of the peptidase C12 family, that produce ubiquitin monomers at ubiquitin C-terminus. UCHL1 replenishes the intracellular store of ubiquitin that is available to tag proteins in their proteasome degraded process [45]. Cerebral ischemia generates reactive lipids, such as cyclopentenone prostaglandins (CyPgs) [46,47], that covalently modify the amino acid cysteine (C152) of UCHL1, reducing its activity [48,49] and promote ischemic damage [50]. Furthermore, UCHL1 activity protects primary neuronal cultures from hypoxia in vitro [51]. These findings suggest that cerebral ischemia can inhibit the regular UCHL1 function, damaging neuronal function and inducing cell death. When studying the association with 4E-BP2, we observed an increase of UCHL1 in the cerebral cortex compared with the CA1 region. This higher association with 4E-BP2 could be a protective condition of this region resistant to ischemia-reperfusion.

The Rho-GDI protein controls the GDP/GTP exchange reaction of Rho proteins and Rho GTPases transport from the endoplasmic reticulum to their site of action on cell membrane [52]. Although Rho-GDI plays a key role in Rho GTPases activity, the underlying mechanisms are still unclear. It has been reported that Rho-GDI is downregulated in neurons in ischemic areas of human patients who have suffered a stroke [31]. Interestingly, we identified Rho-GDI with an important association with 4E-BP2 in the cerebral cortex in response to ischemia-reperfusion that could explain a possible survival mechanism promoting cell repair in this resistant region.

Adenylate kinase 1 (ADK1) isoenzyme, is a sensitive reporter of cellular energy status [53]. Here, ADK1 was identified in the 4E-BP2 interactome, with lower levels of ADK1 in the 4E-BP2 complex in the CA1 region. The higher association with 4E-BP2 in the cerebral cortex could be involved in the recovery of this resistant region after ischemia-reperfusion damage.

The main role of NDKA is nucleoside triphosphates synthesis, other than ATP. Erythropoietin has been reported to increase neuronal expression of NDKA, conferring protection to cortical neurons in ischemia in vitro [54]. Furthermore, extracellular NDKA protein activates mitogen-activated protein kinase (MAPK) pathways in myeloid leukemia cells, which include extracellular signal-regulated kinase (ERK), c-Jun N-terminal kinase (JNK), and p38 signaling molecules [55]. It has also been described that these pathways activation are involved in neuroprotection in cerebral ischemia models [56,57,58,59]. Here, the result of the association of NDKA with 4E-BP2 shows a high interaction in the resistant cortical region induced by ischemia-reperfusion, and a reduction of the NDKA in the 4E-BP2 complex in the vulnerable CA1 region. Therefore, our study supports a contribution of NDKA in the neuroprotection against ischemia-reperfusion through 4E-BP2 interaction.

DRP2 protein is a member of the collapsin response mediator protein (CRMP)/DRP cytosolic phosphoproteins family [30], mostly expressed in brain and involved in axonal growth direction [43]. DRP2 has been described in diverse global and focal ischemia models, identifying different phosphorylated forms, or isoforms of DRP2 with different response to ischemia [30,32,35,60,61,62,63]. Cuadrado et al. [31] described different isoforms of DRP2 in human patients with cerebral infarct. In this study, we identified two spots for DRP2 that could correspond to different phosphorylation status of the protein: DRP2 spot 3 (pI 5.8), with higher levels in the 4E-BP2 interactome in response to ischemia-reperfusion in both cortical and CA1 regions; and DRP2 spot 4 (pI 6.4), with higher association with 4E-BP2 in the cerebral cortex compared with the CA1 region, even though DRP2 levels were lower in ischemia-reperfusion than in the control situation. After these results, DRP2 has emerged as a potential biomarker for ischemia, although the role of its different isoforms in the molecular mechanism of ischemic pathology must be elucidated.

In conclusion, we report for the first time, using immunoprecipitation and a quantitative proteomics analysis, 31 proteins associated with 4E-BP2, which could be considered as a first characterization of the 4E-BP2 interactome. Remarkably, 18 of these proteins presented significant differences after ischemia-reperfusion in an animal model of cerebral ischemia. Two proteins, DRP2 (spot 3) and SOD1, showed increased levels in the 4E-BP2 interactome in response to ischemia-reperfusion and would be potential biomarkers of this pathological condition (Figure 5A). Furthermore, proteins as HSC70, DRP2, enolase 1, UCHL1, ADK1, and particularly NDKA and even more Rho-GDI, exhibit higher association levels to 4E-BP2 in cerebral cortex and could be considered as biomarkers of resilience to cerebral ischemia (Figure 5C). These 4E-BP2-interacting proteins could contribute to the recovery of translation, forming complexes with 4E-BP2 and release “active” eIF4E in the cerebral cortex (Figure 6) being important actors for the protection against ischemic damage in resistant regions. Further studies need to cover the elucidation of the interaction model of these proteins in order to finally identify new therapeutic targets for the diagnosis and treatment of ischemic stroke.

## 4. Materials and Methods

### 4.1. Materials

Rabbit polyclonal anti-4E-BP2 antibodies were from Sigma-Aldrich (Merck KGaA, Darmstadt, Germany) and Cell Signaling Technology (Beverly, MA, USA). Rabbit polyclonal anti-4E-BP1 antibodies were from Cell Signalling Technology. Mouse monoclonal anti-eIF4E antibody was from BD Transduction Laboratories (BD Biosciences, Erembodegen, Belgium). Mouse monoclonal anti-β-tubulin antibody was from Sigma-Aldrich (Merck KGaA, Darmstadt, Germany). All the chemicals used in isoelectric focusing, gel electrophoresis, and DIGE were obtained from Bio-Rad (Madrid, Spain) and Cytiva (formerly GE Healthcare, Barcelona, Spain). All general chemicals were purchased from Sigma-Aldrich (Merck KGaA, Darmstadt, Germany ) unless stated otherwise.

### 4.2. Animal Model of Cerebral Ischemia and Ischemia-Reperfusion

Transient global forebrain ischemia was induced in adult male Wistar rats (10–12 weeks of age, Charles River, L’Arbresle, France), by the standard four-vessel occlusion model, as previously reported [22,23,64]. In brief, animals were anesthetized by intraperitoneal injection with 0.25 mg/kg atropine, 62.5 mg/kg ketamine, and 5 mg/kg diazepam, placed in a stereotaxic frame, and both vertebral arteries were permanently occluded by electrocoagulation. After 24 h, animals were anesthetized with 4% isoflurane for induction and 2–2.5% isoflurane for maintenance (in 80% N_2_/20% O_2_) during the dissection of common carotid arteries and then, both carotid arteries were occluded with small atraumatic clips for 15 min to induce cerebral ischemia. Next, the clips were removed to allow reperfusion. Animal body temperature was controlled and maintained at 37 °C during the surgical procedure. After 3 days of reperfusion (R3d) the animals were sacrificed. Sham control (SHC3d) animals were arranged like the R3d animals without carotid arteries occlusion. A total of 32 animals were used in this study, 16 animals for each SHC3d control and R3d group.

### 4.3. Brain Tissue Samples

Cerebral cortex (C) and hippocampal CA1 region from SHC3d control and R3d ischemic animals were rapidly dissected out under a magnifying glass. Samples were homogenized 1:5 (*w*/*v*) in buffer A (20 mM Tris-HCl, pH 7.5, 140 mM potassium chloride, 5 mM magnesium acetate, 1 mM dithiothreitol (DTT), 2 mM benzamidine, 1 mM EDTA, 2 mM EGTA, 10 µg/mL pepstatin A, 10 µg/mL leupeptin, 10 µg/mL antipain, 20 mM sodium β-glycerophosphate, 20 mM sodium molybdate, 0.2 mM sodium orthovanadate) as previously described [64,65]. The tissue homogenate of each animal was centrifuged at 12,000× *g* for 15 min and the postmitochondrial supernatant (PMS) was collected. All procedures were performed at 4 °C. The PMS sample corresponding to each animal was separately kept at −80 °C until used, and protein concentrations were determined for each sample by the Bradford assay (Bio-Rad, Madrid, Spain).

### 4.4. Immunoprecipitation

4E-BP2 protein was immunoprecipitated in PMS samples from each experimental condition and brain region. For this purpose, PMS samples (300–1200 µg) were pre-clarified incubating (50–100 μL) with Protein G-Agarose 4 (25 µL; 50% slurry, *v/v* in buffer A; ABT) for 1 h on rotating shaker and centrifuged at 16,000× *g* for 5 min. Supernatants were then incubated with rabbit polyclonal anti-4E-BP2 antibody (2–9 µg, Sigma-Aldrich, Merck KGaA, Darmstadt, Germany) overnight and then further incubated with Protein G-Agarose 4 (25 µL; 50% slurry, *v/v* in buffer A) for 1 h. Immunoprecipitates were recovered by centrifugation at 2500× *g* for 5 min, and washed and centrifuged successively three times in buffer A without DTT. All procedures were performed at 4 °C. Finally, immunoprecipitated proteins were eluted from Protein G-Agarose with 8.5 M urea for 30 min at room temperature and subsequent centrifugation at 16,000× *g* for 10 min for 2-D gel electrophoresis analysis, or eluted with loading buffer (60 mM Tris-HCl, pH 6.8, 3% SDS, 2% β-mercaptoethanol, 5% glycerol, 0.0083% bromophenol blue) for one-dimensional SDS-PAGE (see below).

### 4.5. Western Blot Analysis

PMS samples (35 μg) or 4E-BP2 immunoprecipitates were analyzed by one-dimensional SDS-PAGE (12% acrylamide; 2.6% cross-linking) or 2-D gel electrophoresis, and transferred onto PVDF membranes (GE Healthcare, Barcelona, Spain). Membranes were incubated with primary anti-4E-BP1, anti-4E-BP2, anti-eIF4E, or anti-β-tubulin antibodies overnight at 4 °C, membranes were washed and incubated with peroxidase-conjugated secondary anti-mouse or anti-rabbit IgG (both from GE Healthcare, Barcelona, Spain) at room temperature for 1 h, and developed with Clarity ECL reagent (Bio-Rad, Madrid, Spain).

### 4.6. Two-Dimensional Fluorescence Difference in Gel Electrophoresis

4E-BP2 immunoprecipitates from each different experimental sample (SHC3dC, SHC3dCA1, R3dC, and R3dCA1) were analyzed by fluorescence 2-D DIGE. A balanced mixture of 4E-BP2 immunoprecipitates from all groups was used as internal standard for quantitative comparisons [66] as previously described [29]. 4E-BP2 immunoprecipitated samples from control or ischemic groups were labelled with Cy3 or Cy5 dyes, and the internal standard pool was labelled with Cy2 dye, according to the standard CyDye DIGE protocols from GE Healthcare, and labelled samples were kept at −80 °C until used. To avoid possible bias introduced by labelling efficiency, the samples from each experimental group were alternatively labelled with both Cy3 and Cy5 dyes. Before gel electrophoresis analysis, equal protein amounts of 4E-BP2 immunoprecipitated samples from different experimental groups were combined in pairs, and mixed with equal amount of the internal standard and added up to 1% β-mercaptoethanol. Isoelectric focusing (IEF) was performed as first dimension on immobilized (pH 3–10, 11 cm) gradient strips (Bio-Rad) in a flatbed Ettan IPGphor 3 (GE Healthcare, Barcelona, Spain). First, strips were incubated overnight in 200 μL of re-hydration buffer (8 M urea, 2% 3-[(3-cholamidopropyl)dimethylammonio]-1-propanesulfonate (CHAPS), 97 mM DeStreak, 0.2% ampholyte pH 3–10, and 0.001% bromophenol blue) and labelled sample mixture was applied using a loading cup near to the acidic end of the strips. After focusing with a global voltage of 60 kV, strips were equilibrated for 15 min in equilibration buffer (75 mM Tris–HCl pH 8.8, 6 M urea, 30% glycerol, 2% SDS, and 0.004% bromophenol blue). Next, for the second dimension separation, IEF strips with focused proteins were run onto SDS-PAGE mini-gels (12% acrylamide, 2.6% cross-linking) [14,29]. Protein markers (range: 12–225 kDa) and pI standards (range: 3–10) (both from GE Healthcare, Barcelona, Spain) were used to calculate the apparent molecular weight (MW) and pI of resolved proteins. Following electrophoresis, gels were scanned (excluding load sample region) on a Typhoon 9400 (GE Healthcare, Barcelona, Spain) fluorescence imager at 500 V and 100 μm pixel resolution for biological variation analysis. Cy-labelled images were scanned using a 535 nm laser and 650 BP30 emission filter for Cy3 detection, a 633 nm laser and 680 BP30 emission filter for Cy5, and a 488 nm laser and 570 BP30 emission filter for Cy2.

### 4.7. Protein Quantification

DIGE experiments were examined for biological variation analysis (BVA) of the experimental groups (SCH3dC, SHC3dCA1, R3dC, and R3dCA1). DIGE fluorescence images were analyzed for protein abundance variations in the 4E-BP2 immunoprecipitates and the differentially detected proteins between the experimental groups were quantified using PDQuest software (Bio-Rad, Madrid, Spain), and then data were statistically processed using the Microsoft Excel spreadsheet and Prism statistical package (GraphPad Software, San Diego, CA, USA). Proteins (spots) present in all run samples for the same experimental group were considered and selected for protein identification by MALDI-TOF MS.

### 4.8. Protein Identification by MALDI-TOF Mass Spectrometry

Two-dimensional DIGE gels were stained with Coomassie blue for protein identification by MALDI-TOF MS. Protein spots (gel pieces) were manually excised from the gels and processed for in-gel digestion performed as described by Shevchenko et al. with minor modifications [29,67,68]. When necessary, in-gel reduction and protein alkylation were carried out first covering gel pieces with 10 mM DTT in 50 mM ammonium bicarbonate (AB), incubated for 30 min at 56 °C, and after washing with 50 mM AB for 5 min at room temperature, spots were incubated with 55 mM iodoacetamide (IAA) in 50 mM AB for 20 min in dark and washed twice with 50 mM AB followed by 25 mM AB for 5 min. Gel spots were incubated with 50% acetonitrile (ACN) in 25 mM AB twice for 20 min and re-incubated with 100% ACN for 10 min for dehydration and destaining of gel pieces. Then, ACN was completely removed, dry spots were saturated with trypsin (Promega) (30 ng in 25 mM AB) for 45 min on ice and, after removing the excess of trypsin solution, spots were incubated in 25 mM AB overnight at 30 °C. After digestion, 15% ACN were added for 15 min at 37 °C, and next 0.1% trifluoroacetic acid was added for 10 min at room temperature, for peptide extraction. Finally, supernatants were collected for peptide mass fingerprinting (PMF) analysis by MALDI-TOF MS (Autoflex III, Bruker Daltonics, Bremen, Germany) for protein identification, as previously described [29,68]. PMF spectra were obtained in positive mode using an alpha-cyanocinnamic acid matrix and calibrated with a peptide calibration standard mixture (222570, Bruker Daltonics, Bremen, Germany) containing nine standard peptides in a molecular mass range between ~700 and 3500 Da to reach a typical mass measurement accuracy < ±3 ppm. Additionally, when available and for confirmation of protein identity, peptide fragmentation was performed by MS in tandem MALDI LIFT-TOF/TOF [69]. MS data from PMFs spectra and MS/MS data from LIFT TOF/TOF spectra were searched in the SwissProt database using the Mascot database search algorithm (Matrix Science, London, UK) for protein identification. Only one missed tryptic cleavage was allowed and a mass accuracy of 50 ppm was used for all mass searches. MALDI-TOF MS and LIFT TOF/TOF spectra are shown in the Supplementary Material.

### 4.9. Statistical Analysis

Data are represented in arbitrary units (A.U.) and expressed as mean ± SE for the indicated number of experiments. Statistical significance between experimental groups was determined by one-way ANOVA test and, when it was significant, followed by Newman-Keuls post-test for multiple group comparisons, or by Student’s t-test for paired comparisons between the cerebral cortex and CA1 region or between control and ischemic groups. All statistical analyses were performed with Prism software (GraphPad Software, San Diego, CA, USA) and the level of significance was set at α = 0.05.

## Figures and Tables

**Table 1:** 4E-BP2-interacting proteins identified by MALDI-TOF MS with significant changes in response to IR stress.

No.	Protein Identification/Synonyms	Abbreviation ^a^	Accession No. ^b^	Gene Name	Theoretical/Apparent Mass (Da)	Theoretical/Apparent pI	Score ^c^	Coverage (%)	LIFT (Score) ^c,d^
**2**	Heat shock 70 kDa protein 8 /HSP7C	HSC70	P63018	*Hspa8*	71,055/ 72,612	5.37/ 5.5	234	39	2774.32 (126)
**3**	Dihydropyrimidinase-related protein 2/DRP2 /CRMP2 /TOAD64	DRP2	P47942	*Dpysl2*	62,638/ 68,584	5.95/ 5.8	180	38	2377.30 (146)
**4**	Dihydropyrimidinase-related protein 2 /DRP2 /CRMP2 /TOAD64	DRP2	P47942	*Dpysl2*	62,638/ 68,306	5.95/ 6.4	239	56	2070.89 (150)
**9**	Gamma-enolase /Enolase 2 /NSE/ENOG	Enolase 2	P07323	*Eno2*	47,510/ 47,553	5.03/ 5.2	232	61	1358.68 (77)
**10**	Alpha-enolase/Enolase 1 /NNE /ENOA	Enolase 1	P04764	*Eno1*	47,440/ 50,094	6.16/ 6.4	189	61	1541.74 (85)
**12**	Phosphoglycerate kinase 1/PGK1	PGK1	P16617	*Pgk1*	44,909/ 44,365	8.02/ 7.6	72	33	1634.80 (75)
**13**	Glyceraldehyde-3-phosphate dehydrogenase/GAPDH/G3P	G3P	P04797	*Gapdh*	36,090/ 36,027	8.14/ 7.4	53	21	1779.78 (107)
**17**	Fructose-bisphosphate aldolase C/ALDOC	ALDOC	P09117	*Aldoc*	39,658/ 39,979	6.67/ 7.1	234	77	2100.91 (153)
**18**	Pyruvate dehydrogenase E1 component subunit beta/ODPB	ODPB	P49432	*Pdhb*	39,299/ 36,027	6.2/ 5.5	85	31	1747.87 (59)
**25**	Phosphoglycerate mutase 1 /PGAM-B/PGAM1	PGAM1	P25113	*Pgam1*	28,928/ 29,767	6.67/ 6.7	257	72	2135.97 (160)
**26**	Triose-phosphate isomerase/TPIS	TPIS	P48500	*Tpi1*	27,345/ 27,772	6.89/ 7.4	165	55	2206.11 (133)
**28**	Ubiquitin carboxyl-terminal hydrolase isozyme L1 /UCHL1	UCHL1	Q00981	*Uchl1*	25,165/ 28,258	5.14/ 5.2	188	78	1678.83 (119)
**29**	Peroxiredoxin-6/NSGPx /Thiol-specific antioxidant protein /PRDX6	PRDX6	O35244	*Prdx6*	24,860/ 28,752	5.64/ 5.8	130	49	2030.98 (100)
**30**	Protein-L-isoaspartate(D-aspartate) O-methyltransferase /PIMT	PIMT	P22062	*Pcmt1*	24,683/ 27,294	7.14/ 7.2	103	42	1694.85 (45)
**32**	Rho GDP-dissociation inhibitor 1 /Rho-GDI alpha/GDIR1	Rho-GDI	Q5XI73	*Arhgdia*	23,450/ 27,294	5.12/ 5.2	177	70	1650.92 (122)
**33**	Transgelin-3/Neuronal protein NP25/TAGL3	TAGL3	P37805	*Tagln3*	22,657/ 22,164	6.84/ 6.7	95	52	1781.72 (82)
**37**	Adenylate kinase isoenzyme 1 /KAD1	ADK1	P39069	*Ak1*	21,684/ 24,173	7.66/ 7.2	125	63	1495.74 (104)
**39**	Phosphatidylethanolamine-binding protein 1 /PEBP1	PEBP1	P31044	*Pebp1*	20,902/ 23,348	5.48/ 5.5	253	66	1742.89 (76)
**43**	Nucleoside diphosphate kinase A /NDKA	NDKA	Q05982	*Nme1*	17,296/ 17,385	5.96/ 5.8	91	48	1165.62 (39)
**47**	Superoxide dismutase [Cu-Zn], SODC	SOD1	P07632	*Sod1*	16,073/ 17,998	5.88/ 6.1	55	24	1367.79 (108)

^a^, Abbreviations used in this manuscript. ^b^, Accession number in the UniProt database (https://www.uniprot.org/, accessed on 24 June 2021). ^c^, Protein scores > 51 were significant (*p* < 0.05) by peptide mass fingerprint in the Mascot database search algorithm (Matrix Science, London, UK, http://www.matrixscience.com/, accessed on 24 June 2021). ^d^, MALDI LIFT-TOF/TOF identification mode; scores > 24 were significant (*p* < 0.05) by MS/MS ions search performed in Mascot; the fragmented parental peptide and the score (in parenthesis) are indicated.

## Supplementary Materials

The following are available online at https://www.mdpi.com/article/10.3390/ijms221910327/s1. Figure S1: Specific immunoprecipitation of 4E-BP2 in brain tissue samples. Figure S2: Identification of 4E-BP2-interacting proteins. Table S1: 4E-BP2-interacting proteins identified by MALDI-TOF MS without significant changes. Figure S3: Detection of 4E-BP2 and eIF4E proteins in 2-D gel electrophoresis of 4E-BP2 immunoprecipitates. Figure S4: Levels of identified 4E-BP2-interacting proteins without significant changes. Figure S5: Levels and statistical analysis of unidentified 4E-BP2-interacting proteins. Figure S6: Full original images (uncropped) of Western blots of the Figure S1, MALDI-TOF MS and MS/MS spectra.

## References

[1] Fukuyo A., In Y., Ishida T., Tomoo K. (2011). Structural scaffold for eIF4E binding selectivity of 4E-BP isoforms: Crystal structure of eIF4E binding region of 4E-BP2 and its comparison with that of 4E-BP1. J. Pept. Sci..

[2] Haghighat A., Mader S., Pause A., Sonenberg N. (1995). Repression of cap-dependent translation by 4E-binding protein 1: Competition with p220 for binding to eukaryotic initiation factor-4E. EMBO J..

[3] Svitkin Y.V., Herdy B., Costa-Mattioli M., Gingras A.C., Raught B., Sonenberg N. (2005). Eukaryotic translation initiation factor 4E availability controls the switch between cap-dependent and internal ribosomal entry site-mediated translation. Mol. Cell. Biol..

[4] Hay N., Sonenberg N. (2004). Upstream and downstream of mTOR. Genes Dev..

[5] Gkogkas C.G., Khoutorsky A., Ran I., Rampakakis E., Nevarko T., Weatherill D.B., Vasuta C., Yee S., Truitt M., Dallaire P. (2013). Autism-related deficits via dysregulated eIF4E-dependent translational control. Nature.

[6] Saran U., Foti M., Dufour J.F. (2015). Cellular and molecular effects of the mTOR inhibitor everolimus. Clin. Sci..

[7] Chong Z.Z., Yao Q., Li H.H. (2013). The rationale of targeting mammalian target of rapamycin for ischemic stroke. Cell Signal.

[8] Hartman N.W., Lin T.V., Zhang L., Paquelet G.E., Feliciano D.M., Bordey A. (2013). mTORC1 targets the translational repressor 4E-BP2, but not S6 kinase 1/2, to regulate neural stem cell self-renewal in vivo. Cell Rep..

[9] Raught B., Gingras A.C., Mathews M.B., Sonenberg N., Hershey J.W.B. (2007). Signaling to translation initiation. Translational Control in Biology and Medicine.

[10] Ma X.M., Blenis J. (2009). Molecular mechanisms of mTOR-mediated translational control. Nat. Rev. Mol. Cell Biol..

[11] Hidalgo M., Le Bouffant R., Bello V., Buisson N., Cormier P., Beaudry M., Darribere T. (2012). The translational repressor 4E-BP mediates hypoxia-induced defects in myotome cells. J. Cell Sci..

[12] Tsukiyama-Kohara K., Vidal S.M., Gingras A.C., Glover T.W., Hanash S.M., Heng H., Sonenberg N. (1996). Tissue distribution, genomic structure, and chromosome mapping of mouse and human eukaryotic initiation factor 4E-binding proteins 1 and 2. Genomics.

[13] Bidinosti M., Ran I., Sanchez-Carbente M.R., Martineau Y., Gingras A.C., Gkogkas C., Raught B., Bramham C.R., Sossin W.S., Costa-Mattioli M. (2010). Postnatal deamidation of 4E-BP2 in brain enhances its association with raptor and alters kinetics of excitatory synaptic transmission. Mol. Cell.

[14] Ayuso M.I., Martinez-Alonso E., Salvador N., Bonova P., Regidor I., Alcazar A. (2015). Dissociation of eIF4E-binding protein 2 (4E-BP2) from eIF4E independent of Thr37/Thr46 phosphorylation in the ischemic stress response. PLoS ONE.

[15] Chien S., Kandel E.R., Schwartz J.H. (1985). Cerebral blood flow and metabolism. Principles of Neural Science.

[16] Lipton P. (1999). Ischemic cell death in brain neurons. Physiol. Rev..

[17] White B.C., Sullivan J.M., DeGracia D.J., O′Neil B.J., Neumar R.W., Grossman L.I., Rafols J.A., Krause G.S. (2000). Brain ischemia and reperfusion: Molecular mechanisms of neuronal injury. J. Neurol. Sci..

[18] Thilmann R., Xie Y., Kleihues P., Kiessling M. (1986). Persistent inhibition of protein synthesis precedes delayed neuronal death in postischemic gerbil hippocampus. Acta Neuropathol..

[19] Hossmann K.A. (1993). Disturbances of cerebral protein synthesis and ischemic cell death. Prog. Brain Res..

[20] Kirino T. (2000). Delayed neuronal death. Neuropathology.

[21] Pestova T.V., Lorsch J.R., Hellen C.U.T., Mathews M.B., Sonenberg N., Hershey J.M.W. (2007). The mechanism of translation initation in eucaryotes. Translational Control in Biology and Medicine.

[22] Ayuso M.I., Martinez-Alonso E., Cid C., Alonso de Lecinana M., Alcazar A. (2013). The translational repressor eIF4E-binding protein 2 (4E-BP2) correlates with selective delayed neuronal death after ischemia. J. Cereb. Blood Flow Metab..

[23] Pulsinelli W.A., Brierley J.B., Plum F. (1982). Temporal profile of neuronal damage in a model of transient forebrain ischemia. Ann. Neurol..

[24] Wollenhaupt K., Brussow K.P., Albrecht D., Tomek W. (2012). The eIF4E repressor protein 4E-BP2 is merely truncated, despite 4E-BP1 degradation in the porcine uterine tissue during implantation. Mol. Reprod. Dev..

[25] Schaller B., Graf R. (2004). Cerebral ischemia and reperfusion: The pathophysiologic concept as a basis for clinical therapy. J. Cereb. Blood Flow Metab..

[26] Burda J., Martin M.E., Garcia A., Alcazar A., Fando J.L., Salinas M. (1994). Phosphorylation of the alpha subunit of initiation factor 2 correlates with the inhibition of translation following transient cerebral ischaemia in the rat. Biochem. J..

[27] DeGracia D.J., Neumar R.W., White B.C., Krause G.S. (1996). Global brain ischemia and reperfusion: Modifications in eukaryotic initiation factors associated with inhibition of translation initiation. J. Neurochem..

[28] DeGracia D.J., Hu B.R. (2007). Irreversible translation arrest in the reperfused brain. J. Cereb. Blood Flow Metab..

[29] Cid C., Garcia-Bonilla L., Camafeita E., Burda J., Salinas M., Alcazar A. (2007). Proteomic characterization of protein phosphatase 1 complexes in ischemia-reperfusion and ischemic tolerance. Proteomics.

[30] Chen A., Liao W.P., Lu Q., Wong W.S., Wong P.T. (2007). Upregulation of dihydropyrimidinase-related protein 2, spectrin alpha II chain, heat shock cognate protein 70 pseudogene 1 and tropomodulin 2 after focal cerebral ischemia in rats—A proteomics approach. Neurochem. Int..

[31] Cuadrado E., Rosell A., Colome N., Hernandez-Guillamon M., Garcia-Berrocoso T., Ribo M., Alcazar A., Ortega-Aznar A., Salinas M., Canals F. (2010). The proteome of human brain after ischemic stroke. J. Neuropathol. Exp. Neurol..

[32] Koh P.O. (2010). Proteomic analysis of focal cerebral ischemic injury in male rats. J. Vet. Med. Sci..

[33] Koh P.O. (2011). Identification of proteins differentially expressed in cerebral cortexes of Ginkgo biloba extract (EGb761)-treated rats in a middle cerebral artery occlusion model—A proteomics approach. Am. J. Chin. Med..

[34] Hori M., Nakamachi T., Rakwal R., Shibato J., Ogawa T., Aiuchi T., Tsuruyama T., Tamaki K., Shioda S. (2012). Transcriptomics and proteomics analyses of the PACAP38 influenced ischemic brain in permanent middle cerebral artery occlusion model mice. J. Neuroinflamm..

[35] Campos-Martorell M., Salvador N., Monge M., Canals F., Garcia-Bonilla L., Hernandez-Guillamon M., Ayuso M.I., Chacon P., Rosell A., Alcazar A. (2014). Brain proteomics identifies potential simvastatin targets in acute phase of stroke in a rat embolic model. J. Neurochem..

[36] Mayer M.P., Bukau B. (2005). Hsp70 chaperones: Cellular functions and molecular mechanism. Cell Mol. Life Sci..

[37] Wang X., Wang Q., Lin H., Li S., Sun L., Yang Y. (2013). HSP72 and gp96 in gastroenterological cancers. Clin. Chim. Acta.

[38] Xilouri M., Stefanis L. (2016). Chaperone mediated autophagy in aging: Starve to prosper. Ageing Res. Rev..

[39] Kawagoe J., Abe K., Sato S., Nagano I., Nakamura S., Kogure K. (1992). Distributions of heat shock protein (HSP) 70 and heat shock cognate protein (HSC) 70 mRNAs after transient focal ischemia in rat brain. Brain Res..

[40] Aoki M., Abe K., Kawagoe J., Sato S., Nakamura S., Kogure K. (1993). Temporal profile of the induction of heat shock protein 70 and heat shock cognate protein 70 mRNAs after transient ischemia in gerbil brain. Brain Res..

[41] Brea D., Agulla J., Staes A., Gevaert K., Campos F., Sobrino T., Blanco M., Davalos A., Castillo J., Ramos-Cabrer P. (2015). Study of Protein Expression in Peri-Infarct Tissue after Cerebral Ischemia. Sci. Rep..

[42] Pancholi V. (2001). Multifunctional alpha-enolase: Its role in diseases. Cell Mol. Life Sci..

[43] Poon H.F., Vaishnav R.A., Getchell T.V., Getchell M.L., Butterfield D.A. (2006). Quantitative proteomics analysis of differential protein expression and oxidative modification of specific proteins in the brains of old mice. Neurobiol. Aging.

[44] Doran J.F., Jackson P., Kynoch P.A., Thompson R.J. (1983). Isolation of PGP 9.5, a new human neurone-specific protein detected by high-resolution two-dimensional electrophoresis. J. Neurochem..

[45] Osaka H., Wang Y.L., Takada K., Takizawa S., Setsuie R., Li H., Sato Y., Nishikawa K., Sun Y.J., Sakurai M. (2003). Ubiquitin carboxy-terminal hydrolase L1 binds to and stabilizes monoubiquitin in neuron. Hum. Mol. Genet..

[46] Ahmad M., Dar N.J., Bhat Z.S., Hussain A., Shah A., Liu H., Graham S.H. (2014). Inflammation in ischemic stroke: Mechanisms, consequences and possible drug targets. CNS Neurol. Disord. Drug Targets.

[47] Figueiredo-Pereira M.E., Rockwell P., Schmidt-Glenewinkel T., Serrano P. (2015). Neuroinflammation and J2 prostaglandins: Linking impairment of the ubiquitin-proteasome pathway and mitochondria to neurodegeneration. Front Mol. Neurosci..

[48] Kondo M., Shibata T., Kumagai T., Osawa T., Shibata N., Kobayashi M., Sasaki S., Iwata M., Noguchi N., Uchida K. (2002). 15-Deoxy-Delta (12,14)-prostaglandin J (2): The endogenous electrophile that induces neuronal apoptosis. Proc. Natl. Acad. Sci. USA.

[49] Liu H., Li W., Rose M.E., Hickey R.W., Chen J., Uechi G.T., Balasubramani M., Day B.W., Patel K.V., Graham S.H. (2015). The point mutation UCH-L1 C152A protects primary neurons against cyclopentenone prostaglandin-induced cytotoxicity: Implications for post-ischemic neuronal injury. Cell Death Dis..

[50] Graham S.H., Liu H. (2017). Life and death in the trash heap: The ubiquitin proteasome pathway and UCHL1 in brain aging, neurodegenerative disease and cerebral Ischemia. Ageing Res. Rev..

[51] Liu H., Li W., Ahmad M., Miller T.M., Rose M.E., Poloyac S.M., Uechi G., Balasubramani M., Hickey R.W., Graham S.H. (2011). Modification of ubiquitin-C-terminal hydrolase-L1 by cyclopentenone prostaglandins exacerbates hypoxic injury. Neurobiol. Dis..

[52] Dovas A., Couchman J.R. (2005). RhoGDI: Multiple functions in the regulation of Rho family GTPase activities. Biochem. J..

[53] Dzeja P.P., Bast P., Pucar D., Wieringa B., Terzic A. (2007). Defective metabolic signaling in adenylate kinase AK1 gene knock-out hearts compromises post-ischemic coronary reflow. J. Biol. Chem..

[54] Teoh J., Boulos S., Chieng J., Knuckey N.W., Meloni B.P. (2014). Erythropoietin increases neuronal NDPKA expression, and NDPKA up-regulation as well as exogenous application protects cortical neurons from in vitro ischemia-related insults. Cell Mol. Neurobiol..

[55] Okabe-Kado J., Kasukabe T., Honma Y., Kobayashi H., Maseki N., Kaneko Y. (2009). Extracellular NM23 protein promotes the growth and survival of primary cultured human acute myelogenous leukemia cells. Cancer Sci..

[56] Huang C.Y., Liou Y.F., Chung S.Y., Lin W.Y., Jong G.P., Kuo C.H., Tsai F.J., Cheng Y.C., Cheng F.C., Lin J.Y. (2010). Role of ERK signaling in the neuroprotective efficacy of magnesium sulfate treatment during focal cerebral ischemia in the gerbil cortex. Chin. J. Physiol..

[57] Karmarkar S.W., Bottum K.M., Krager S.L., Tischkau S.A. (2011). ERK/MAPK is essential for endogenous neuroprotection in SCN2.2 cells. PLoS ONE.

[58] Wang S., Guo S.X., Dai Z.G., Dong X.W., Liu Y., Jiang S., Wang Z.P. (2011). Dual isoflurane-induced preconditioning improves neuroprotection in rat brain in vitro and the role of extracellular signal—Regulated protein kinase. Chin. Med. Sci. J..

[59] Jiang S.Y., Zou Y.Y., Wang J.T. (2012). p38 mitogen-activated protein kinase-induced nuclear factor kappa-light-chain-enhancer of activated B cell activity is required for neuroprotection in retinal ischemia/reperfusion injury. Mol. Vis..

[60] Zhou Y., Bhatia I., Cai Z., He Q.Y., Cheung P.T., Chiu J.F. (2008). Proteomic analysis of neonatal mouse brain: Evidence for hypoxia- and ischemia-induced dephosphorylation of collapsin response mediator proteins. J. Proteome Res..

[61] Teilum M., Krogh M., Wieloch T., Mattiasson G. (2007). Hypothermia affects translocation of numerous cytoplasmic proteins following global cerebral ischemia. J. Proteome Res..

[62] Kobeissy F.H., Ottens A.K., Zhang Z., Liu M.C., Denslow N.D., Dave J.R., Tortella F.C., Hayes R.L., Wang K.K. (2006). Novel differential neuroproteomics analysis of traumatic brain injury in rats. Mol. Cell Proteomics.

[63] Kwan J.A., Schulze C.J., Wang W., Leon H., Sariahmetoglu M., Sung M., Sawicka J., Sims D.E., Sawicki G., Schulz R. (2004). Matrix metalloproteinase-2 (MMP-2) is present in the nucleus of cardiac myocytes and is capable of cleaving poly (ADP-ribose) polymerase (PARP) in vitro. FASEB J..

[64] Martin de la Vega C., Burda J., Nemethova M., Quevedo C., Alcazar A., Martin M.E., Danielisova V., Fando J.L., Salinas M. (2001). Possible mechanisms involved in the down-regulation of translation during transient global ischaemia in the rat brain. Biochem. J..

[65] Garcia-Bonilla L., Cid C., Alcazar A., Burda J., Ayuso I., Salinas M. (2007). Regulatory proteins of eukaryotic initiation factor 2-alpha subunit (eIF2 alpha) phosphatase, under ischemic reperfusion and tolerance. J. Neurochem..

[66] Alban A., David S.O., Bjorkesten L., Andersson C., Sloge E., Lewis S., Currie I. (2003). A novel experimental design for comparative two-dimensional gel analysis: Two-dimensional difference gel electrophoresis incorporating a pooled internal standard. Proteomics.

[67] Shevchenko A., Tomas H., Havlis J., Olsen J.V., Mann M. (2006). In-gel digestion for mass spectrometric characterization of proteins and proteomes. Nat. Protoc..

[68] Martinez-Alonso E., Alcazar P., Camafeita E., Fernandez-Lucas M., Ruiz-Roso G., Alcazar A. (2020). Proteomic analysis of plasma proteins of high-flux haemodialysis and on-line haemodiafiltration patients reveals differences in transthyretin levels related with anaemia. Sci. Rep..

[69] Suckau D., Resemann A., Schuerenberg M., Hufnagel P., Franzen J., Holle A. (2003). A novel maldi lift-tof/tof mass spectrometer for proteomics. Anal. Bioanal. Chem..

