# Supplementary material for: Differential Association of 4E-BP2-Interacting Proteins Is Related to Selective Delayed Neuronal Death after Ischemia
Source: Int J Mol Sci. 2021 Sep 25;22(19):10327. doi: 10.3390/ijms221910327 (PMC8509075; doi:10.3390/ijms221910327)
Supplement: Supplementary file 1 [file ijms-22-10327-s001.zip › ijms-1369112-supplementary.pdf]

Supplementary Material

Differential Association of 4E-BP2–Interacting Proteins Is Related to Selective Delayed Neuronal Death after Ischemia

Emma Martínez-Alonso[1,2], Natalia Guerra-Pérez[1,3], Alejandro Escobar-Peso[1], Ignacio Regidor[4], Jaime Masjuan[5,6] and Alberto Alcázar[1,2,*]

[1] Department of Research, Hospital Universitario Ramón y Cajal, IRYCIS, Ctra. Colmenar km 9.1, Madrid 28034, Spain; emma.martinez@hrc.es (E.M.-A.); natalgue@ucm.es (N.G.-P.); alejandro.escobar@hrc.es (A.E.-P.); alberto.alcazar@hrc.es (A.A.)

[2] Proteomics Unit. Hospital Universitario Ramón y Cajal, IRYCIS, Ctra. Colmenar km 9.1, Madrid 28034, Spain

[3] Department of Genetics, Physiology and Microbiology, Faculty of Biological Sciences, Complutense University of Madrid, Av. Complutense, 28040 Madrid, Spain

[4] Department of Neurophysiology, Hospital Universitario Ramón y Cajal, IRYCIS, Ctra. Colmenar km 9.1, Madrid 28034, Spain; ignacio.regidor@salud.madrid.org (I.R.)

[5] Department of Neurology, Hospital Universitario Ramón y Cajal, IRYCIS, Ctra. Colmenar km 9.1, Madrid 28034, Spain; jaime.masjuan@salud.madrid.org (J.M.)

[6] Department of Neurology, Facultad de Medicina, Universidad de Alcalá, Ctra. Madrid-Barcelona km 33.6, Alcalá de Henares 28871, Spain

* Correspondence: alberto.alcazar@hrc.es

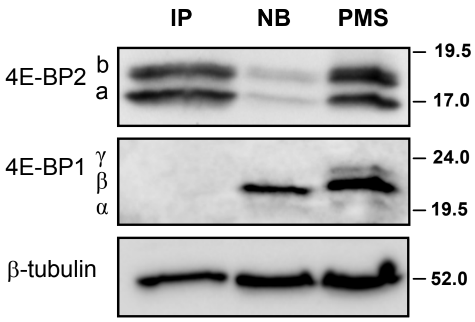

Figure S1. Specific immunoprecipitation of 4E-BP2 in brain tissue samples. Postmitochondrial supernatant (PMS) samples of cerebral cortex from 3-day sham control animals (SHC3dC) were immunoprecipitated with anti–4E-BP2 antibody (from Sigma-Aldrich) and subsequent incubation with Protein G-Agarose for 4E-BP2 immunoprecipitation (IP); the non-bound fraction to Protein G-Agarose was recovered as NB fraction. IP and NB samples, and the immunoprecipitated PMS (lanes IP, NB and PMS, respectively) were analyzed by western blotting with anti–4E-BP2 antibody (from Cell Signalling), and after stripping, re-blotted with anti–4E-BP1 antibody. The figure shows representative blots of 4E-BP2 (upper image) and 4E-BP1 (middle image), detecting the subunits *a* and *b*, and α, β and γ, of 4E-BP2 and 4E-BP1 proteins, respectively. No 4E-BP1 was detected in 4E-BP2 immunoprecipitates (lane IP), but was detected in the non-bound fraction (lane NB). On the contrary, 4E-BP2 was almost undetected in the non-bound fraction. Blot developed with anti–β-tubulin antibody is shown as loading control (lower image). Numbers on the right indicate the apparent molecular weight (in kDa). Full original images of the western blots are shown at the end of this Supplementary Material (Figure S6).

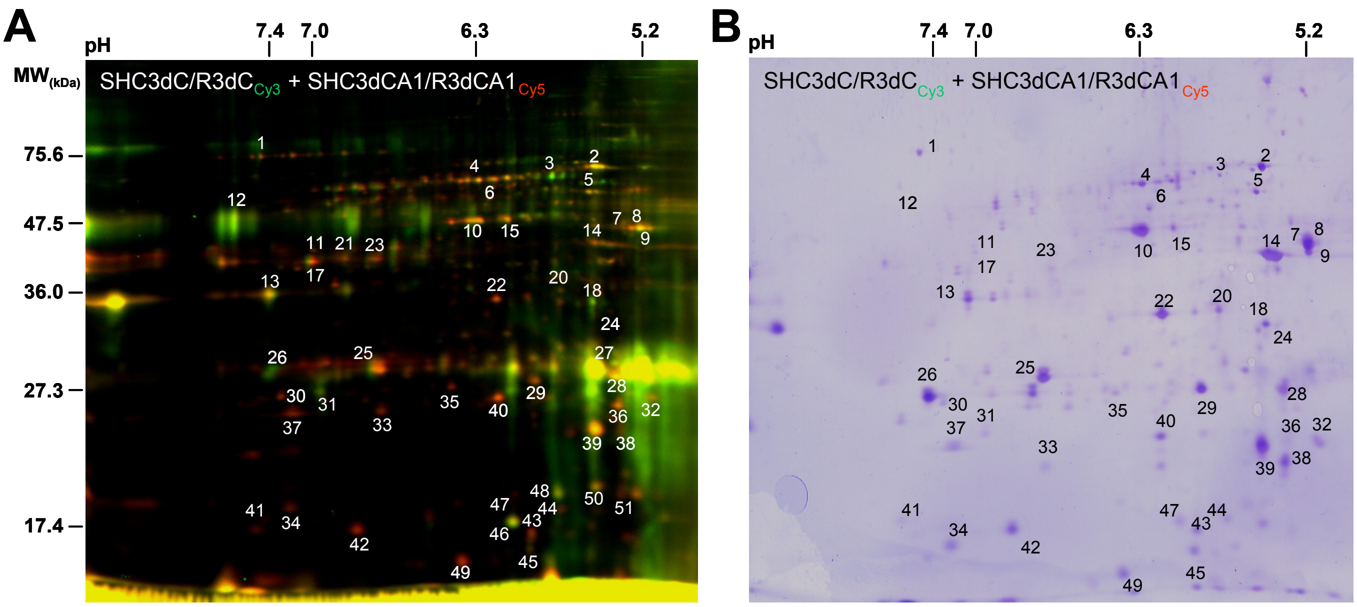

Figure S2. Identification of 4E-BP2–interacting proteins. (A) Preparative 2-D DIGE scanned image shows the overlay of the 4E-BP2 immunoprecipitates of PMS samples (1200 μg) from the cerebral cortex (SHC3dC and R3dC), labelled with Cy3 (green), and from the CA1 region (SHC3dCA1 and R3dCA1) labelled with Cy5 (red). After scanning, the gel was stained with Coomassie blue (B). Protein spots were matched between the fluorescence scanned image (A) and the Coomassie stained gel (B). Matched 4E-BP2–associated proteins (spots) were tagged with numbers and subsequently excised for identification by MALDI-TOF/TOF MS. Gel images are full original images; the horizontal axis represents pH and vertical axis represents molecular weight (MW, in kDa).

Table S1. 4E-BP2–interacting proteins identified by MALDI-TOF MS without significant changes.

No.	Protein Identification /Synonyms	Abbreviation[a]	Accession No.[b]	Gene Name	Theoretical / Apparent Mass (Da)	Theoretical/ Apparent pI	Score[c]	Coverage (%)	LIFT (Score)[c,d]
1	Aconitate hydratase /Aconitase /ACON	Aconitase	Q9ER34	*Aco2*	86121/ 75634	7.87/ 7.4	216	31	1667.78 (109)
7	Tubulin alpha-1B chain /Tubulin alpha-2 chain /TBA1B	Tubulin α1B	Q6P9V9	*Tuba1b*	50804/ 50970	4.94/ 5.2	112	41	2415.17 (97)
14	Actin, cytoplasmic 1 /Beta-actin/ACTB	β-Actin	P60711	*Actb*	42052/ 45931	5.29/ 5.4	168	55	2215.05 (144)
20	L-lactate dehydrogenase B chain /LDH-H /LDHB	LDHB	P42123	*Ldhb*	36874/ 37299	5.7/ 5.6	52	18	2296.16 (60)
22	Malate dehydrogenase, cytoplasmic /MDHC	MDHC	O88989	*Mdh1*	36631/ 37299	6.16/ 6.1	70	25	1706.85 (61)
31	GTP-binding nuclear protein Ran/Ras–related nuclear protein /RAN	RAN	P62828	*Ran*	24579/ 24596	7.01/ 7.0	113	34	1784.89 (83)
36	Peroxiredoxin-2 /PRDX2	PRDX2	P35704	*Prdx2*	21941/ 22947	5.34/ 5.4	143	68	1211.68 (56)
40	Protein DJ-1 /PARK7	PARK7	O88767	*Park7*	20190/ 25464	6.32/ 6.3	125	62	1230.72 (58)
42	Nucleoside diphosphate kinase B /NDKB	NDKB	P19804	*Nme2*	17386/ 17385	6.92/ 6.9	136	73	1175.67 (64)
44	Stathmin /Prosolin/pp17 /STMN1	STMN1	P13668	*Stmn1*	17278/ 17998	5.76/ 5.7	38	16	1388.70 (55)
49	Histidine triad nucleotide–binding protein 1 /HINT1	HINT	P62959	*Hint1*	13882/ 14871	6.36/ 6.2	98	47	2545.25 (156)

[a], Abbreviations used in this manuscript. [b], Accession number in the UniProt database (https://www.uniprot.org/, accessed on 24 June 2021). [c], Protein scores > 51 were significant ($p<0.05$) by peptide mass fingerprint in the MASCOT database search algorithm (Matrix Science, London, UK, http://www.matrixscience.com/, accessed on 24 June 2021). [d], MALDI LIFT-TOF/TOF identification mode; scores > 24 were significant ($p<0.05$) by MS/MS ions search performed in MASCOT; the fragmented parental peptide and the score (in parenthesis) are indicated.

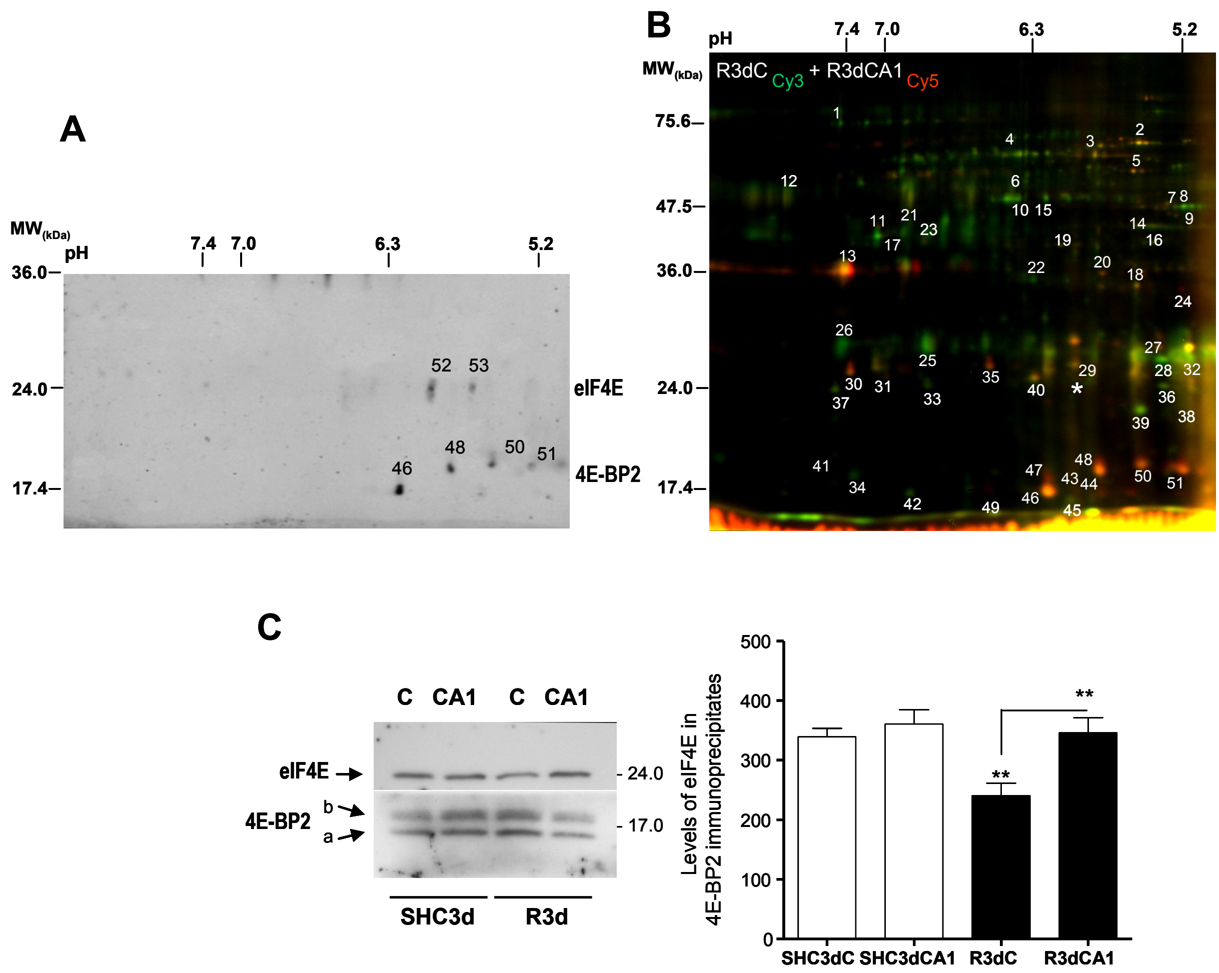

Figure S3. Detection of 4E-BP2 and eIF4E proteins in 2-D gel electrophoresis of 4E-BP2 immunoprecipitates. (A) 4E-BP2 immunoprecipitate of the cerebral cortex from control and ischemic samples (SHC3dC and R3dC; 600 μg) were resolved by 2-D gel electrophoresis run in parallel to the experiment shown in Figure 2, and incubated, after gel blotting, with anti–eIF4E antibody and anti–mouse IRDye 680LT-conjugated secondary antibody, and with anti–4E-BP2 antibody and anti–rabbit IRDye 800CW-conjugated antibody, for simultaneous detection of eIF4E and 4E-BP2, respectively. Blotted membrane was cut in 36-kDa region before antibody incubation. Images (A) and (B) are full original images; the horizontal axis represents pH and the vertical axis represents molecular weight (MW, in kDa). 4E-BP2 protein was identified as protein spots 46, 48, 50 and 51 by the exact matching with 2-D DIGE gel from Figure 2A (B). eIF4E protein spots (52 and 53) were identified with the anti–eIF4E antibody (A), and they were absent in the DIGE gel (B). In (B), the position of spot 52 is marked by an asterisk. (C) eIF4E and 4E-BP2 detection by western blotting in 4E-BP2 immunoprecipitates of the cerebral cortex, C, and CA1 region, from SHC3d control and R3d ischemic samples using the same primary antibodies as in (A) and peroxidase-conjugated secondary antibodies. The figure shows representative blots of eIF4E (upper image) and 4E-BP2 (lower image), detecting the subunits *a* and *b* of 4E-BP2. Numbers on the right indicate the apparent molecular weight (in kDa). Full original images of western blots are shown, and they were cut in ~21-kDa region to avoid immunoglobulin light chain signal from immunoprecipitation procedure The bar graph show the levels of eIF4E quantified in the immunoprecipitates. Error bars indicate SE. Statistical significance were performed by Newman-Keuls post-test (**$p < 0.01$) after significant ANOVA ($p < 0.05$), compared with control, or between the cerebral cortex and CA1 samples (line). The vertical axis indicates the quantification values in arbitrary units.

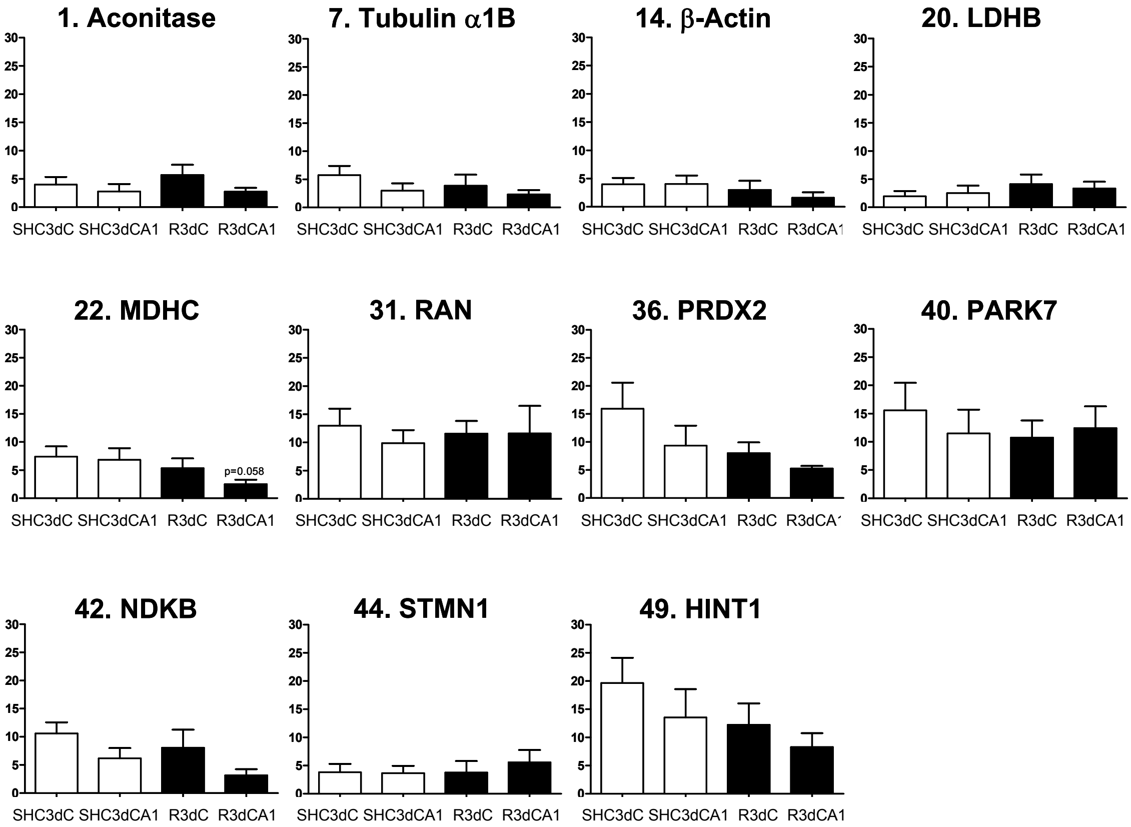

Figure S4. Levels of identified 4E-BP2–interacting proteins without significant changes. Bar graphs show the 4E-BP2–associated protein levels as described in Figure S4. Statistical analysis was not significant (ANOVA, $p > 0.05$). The vertical axis indicates the quantification values of the spot intensity in arbitrary units. Graphs are numbered and named according to the proteins listed in Table 1.

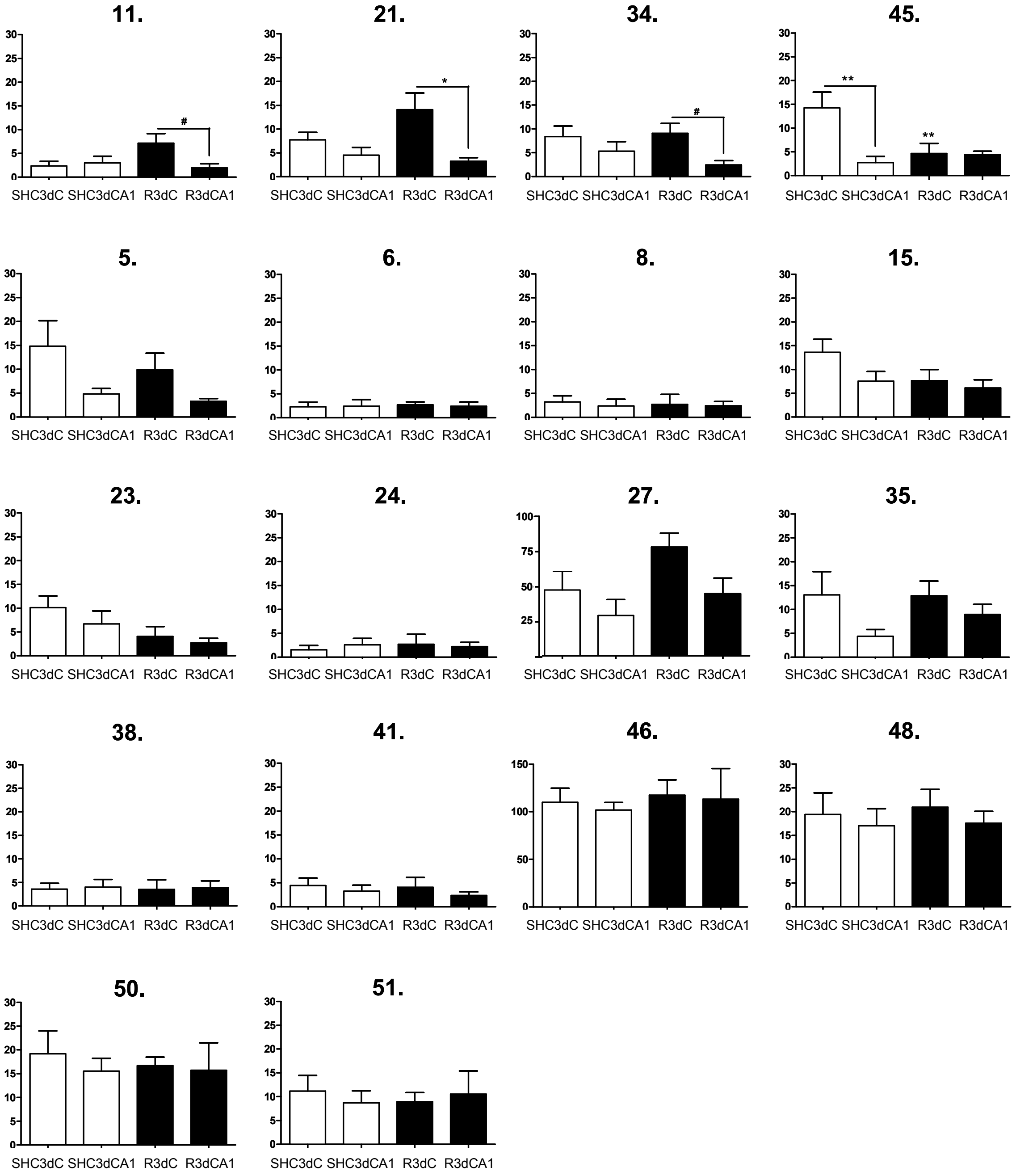

Figure S5. Levels and statistical analysis of unidentified 4E-BP2–interacting proteins. Bar graphs show the 4E-BP2–associated protein levels as described in Figure S4. Statistical analysis was performed as in Figure S4. Only proteins 11, 21, 34 and 45 were significant (ANOVA, $p < 0.05$) and post-test were performed by Newman-Keuls (*$p < 0.05$; **$p < 0.01$) or by Student's t-test (#$p < 0.05$; ##$p < 0.01$), compared with their respective control, or between the cerebral cortex and CA1 samples (indicated by lines). The vertical axis indicates the quantification values of the spot intensity in arbitrary units. Graphs are numbered and named according to the proteins listed in Table S1. Note that proteins 46, 48, 50 and 51 were later identified as 4E-BP2 protein (Figure S3).

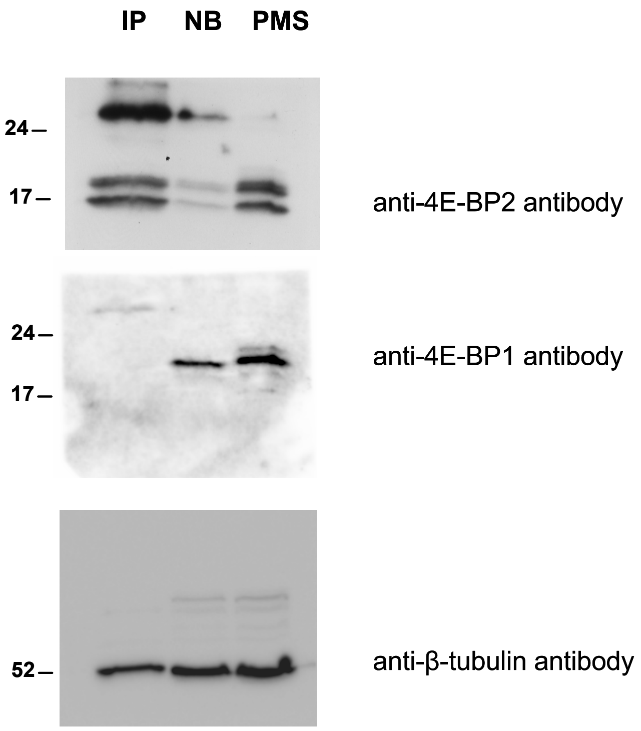

Figure S6. Full original images (uncropped) of western blots of the Figure S1, incubated with anti–4E-BP2, anti–4E-BP1 and anti–β-tubulin antibodies. Numbers on the left indicate the apparent molecular weight (in kDa). In the upper image, the band above 24 kDa corresponds to immunoglobulin light chains from immunoprecipitation procedure.

MALDI-TOF MS spectra

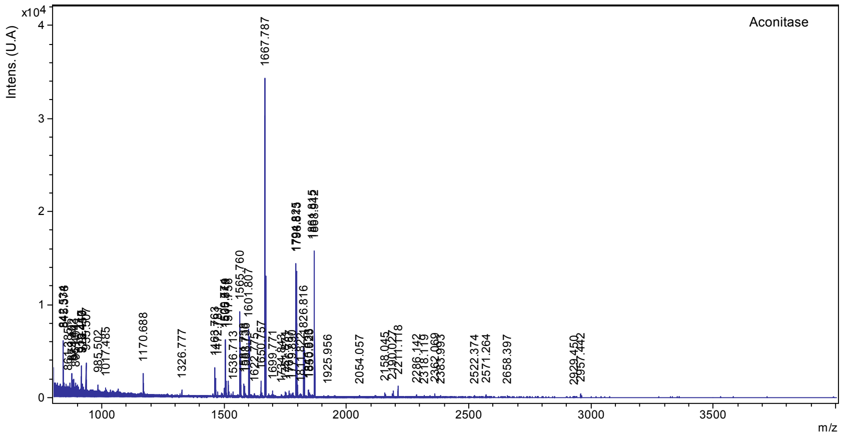

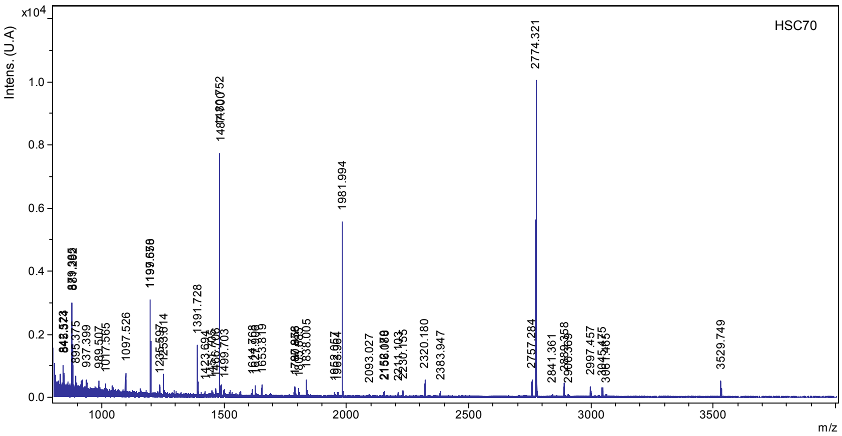

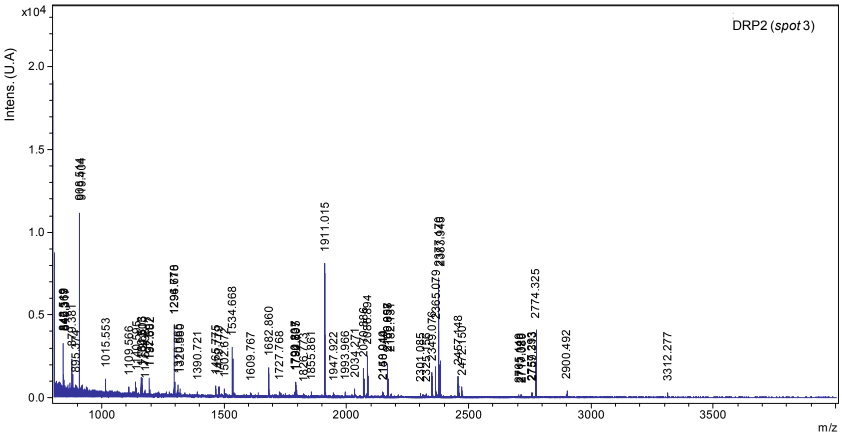

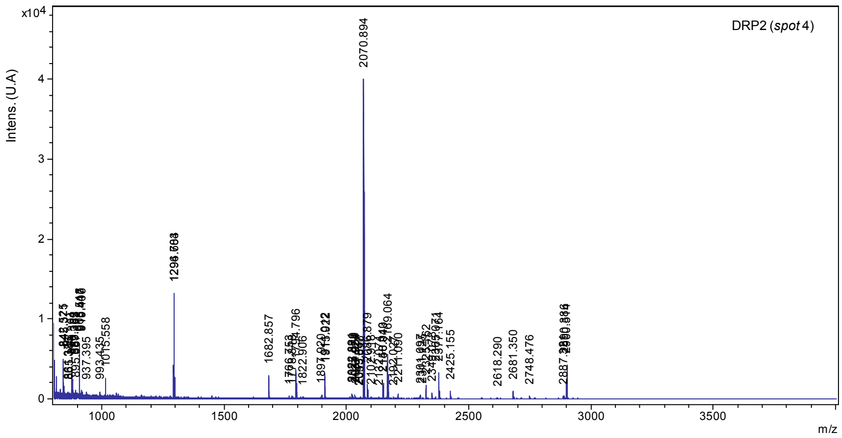

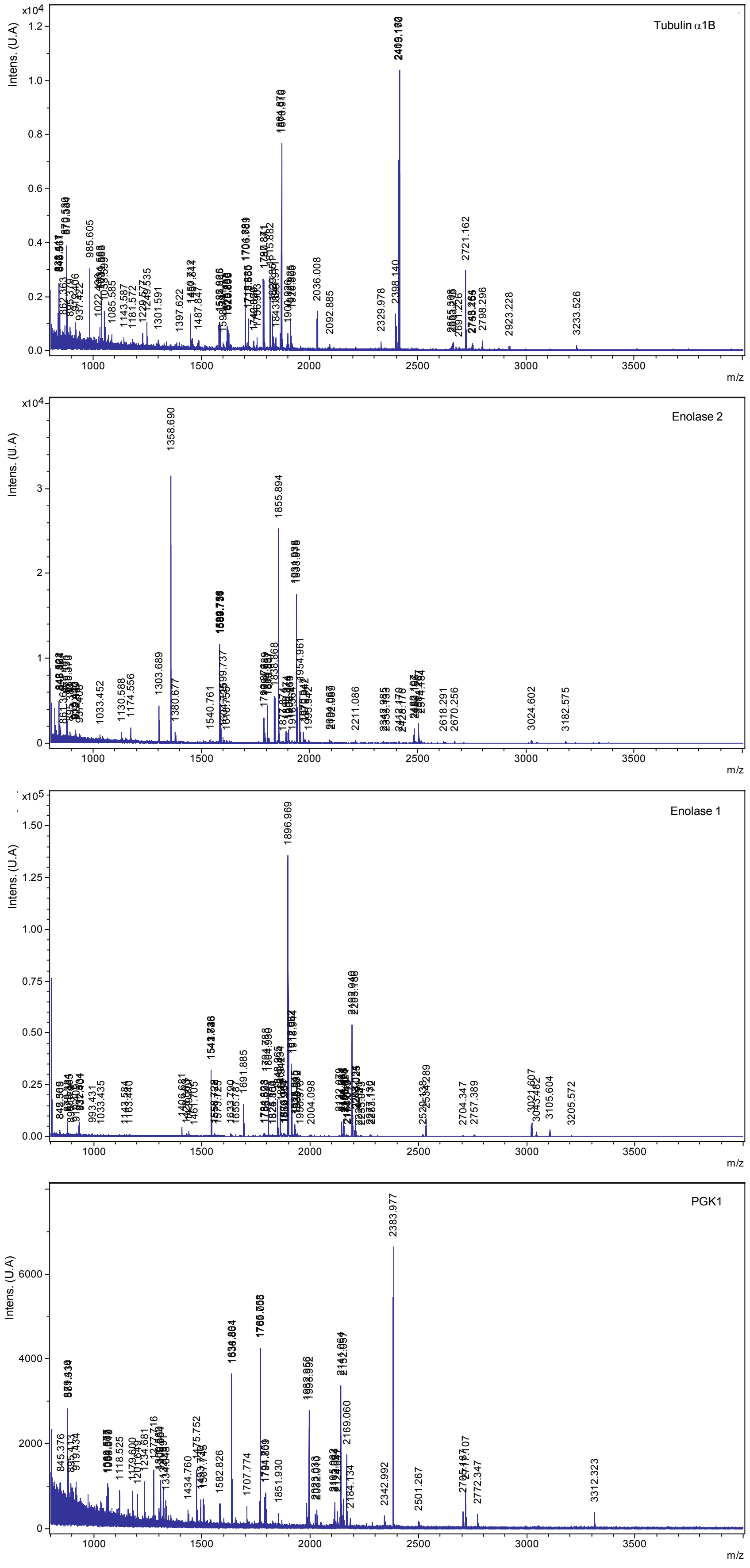
Tubulin α1B
Enolase 2
Enolase 1
PGK1
Intens. (U.A)
m/z

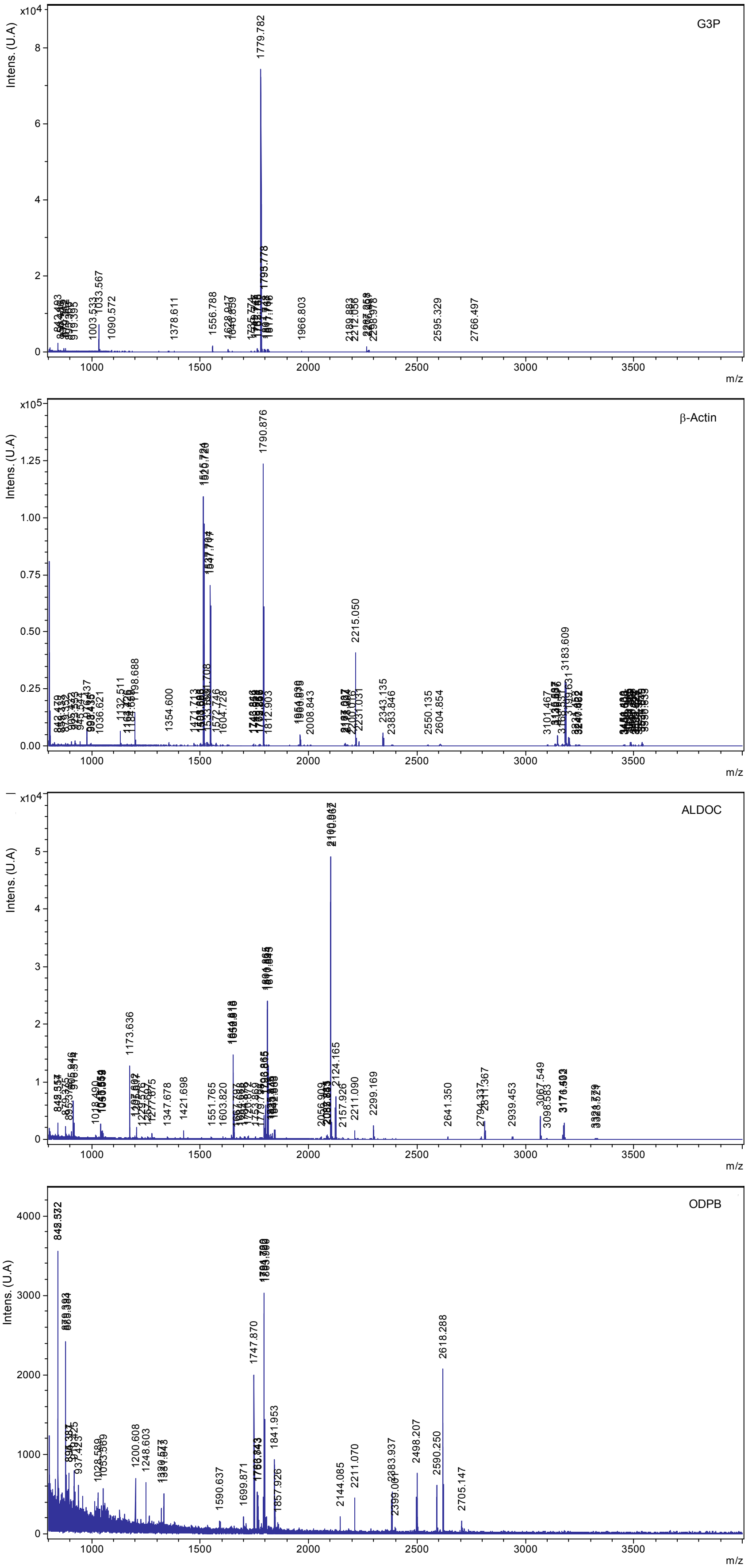
G3P
Intens. (U.A)
x10^4
1779.782
1795.778
1033.567
β-Actin
x10^5
1790.876
2215.050
3183.609
ALDOC
2124.165
1173.636
ODPB
1747.870
2618.288
1841.953
m/z

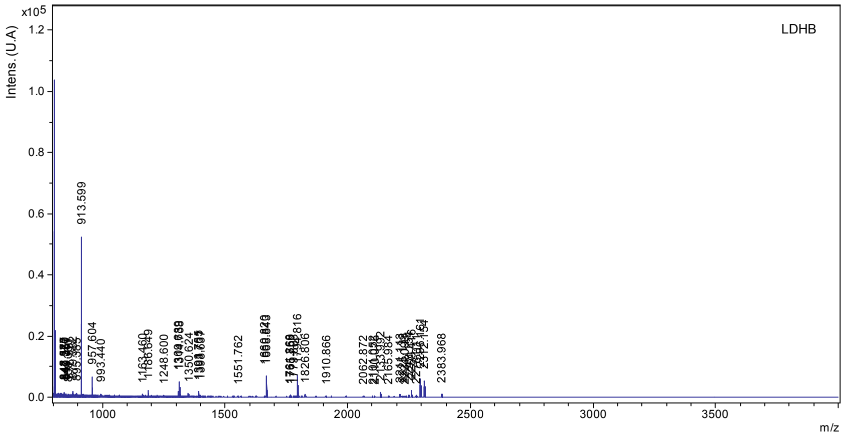
LDHB
Intens. (U.A)
x10^5
m/z
913.599

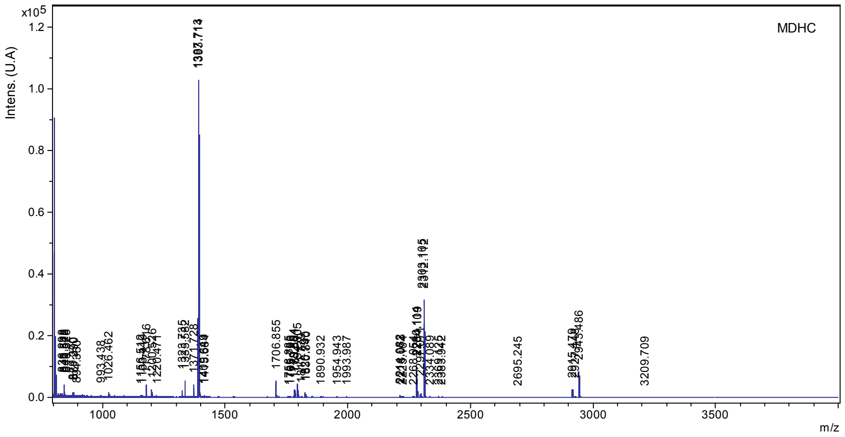
MDHC
Intens. (U.A)
x10^5
m/z

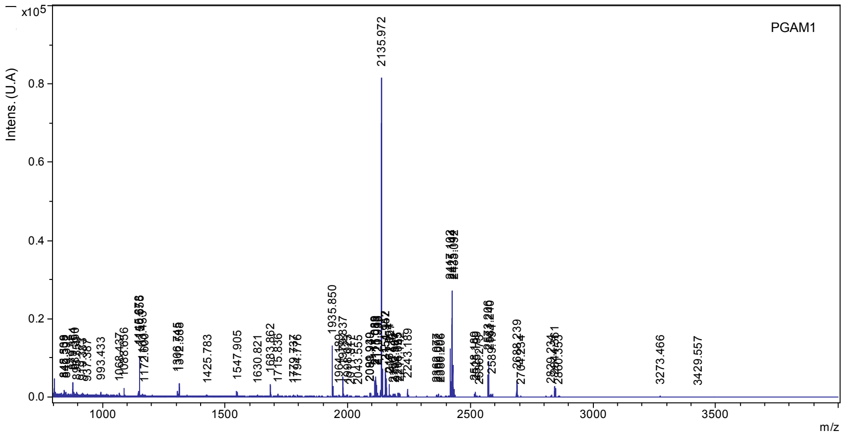
PGAM1
Intens. (U.A)
x10^5
m/z
2135.972

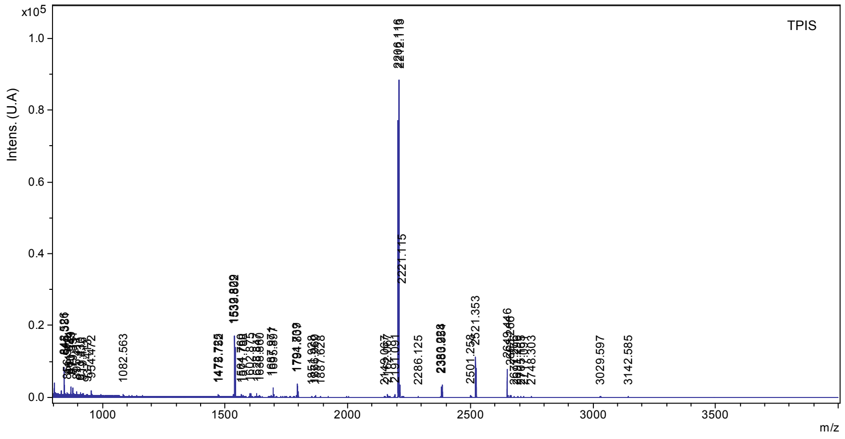
TPIS
Intens. (U.A)
x10^5
m/z
2221.115

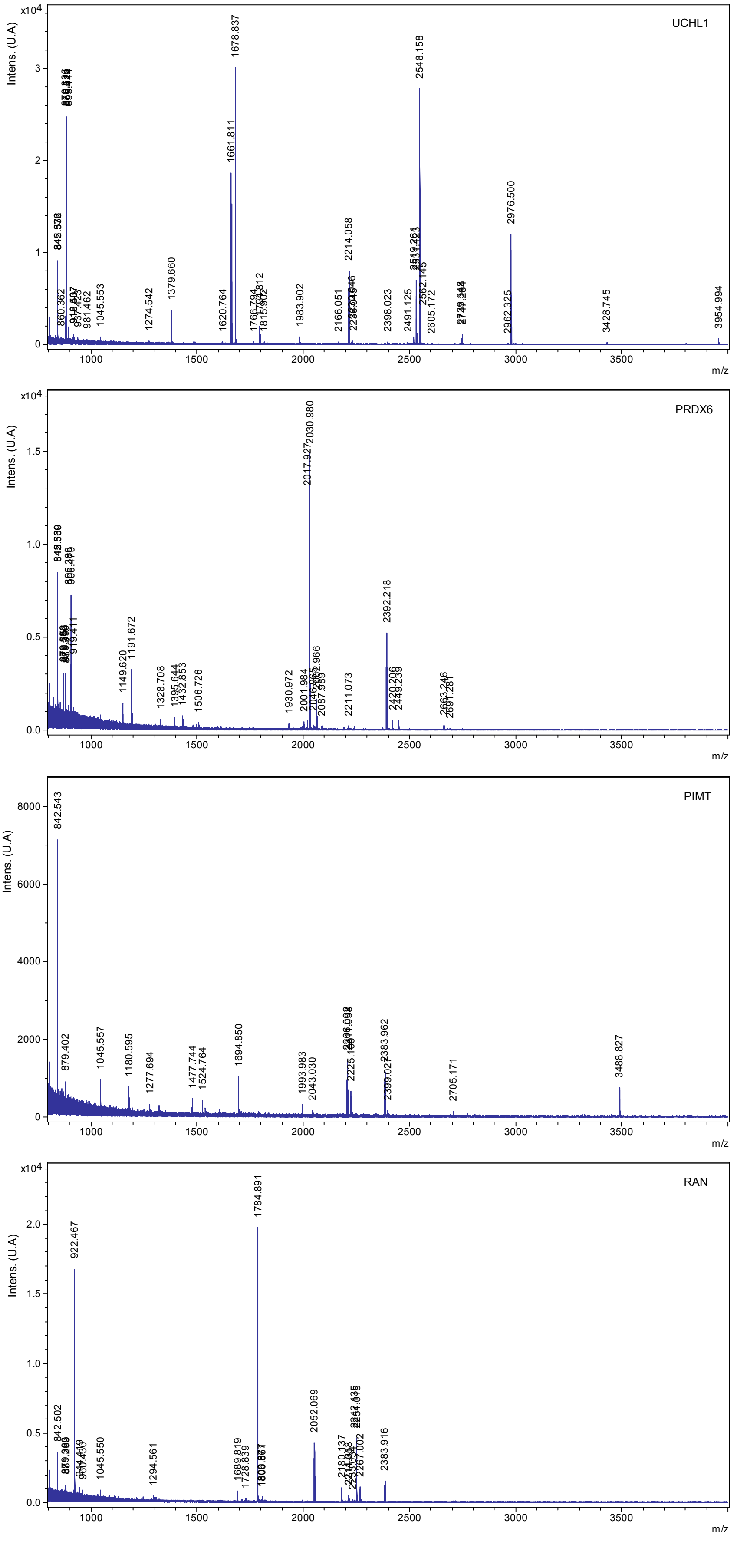
UCHL1
PRDX6
PIMT
RAN
Intens. (U.A)
m/z
x10^4
1678.837
1661.811
2548.158
2976.500
2214.058
1379.660
2030.980
2392.218
1191.672
1149.620
842.543
1694.850
3488.827
2383.962
1045.557
1180.595
1784.891
922.467
2052.069
2383.916
842.502

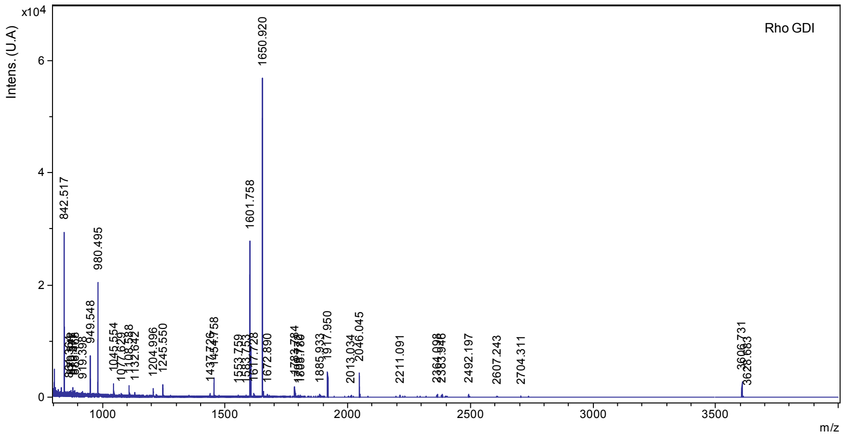
Rho GDI
Intens. (U.A)
x10^4
m/z
1650.920
1601.758
842.517
980.495
949.548
2046.045
1917.950
3606.731

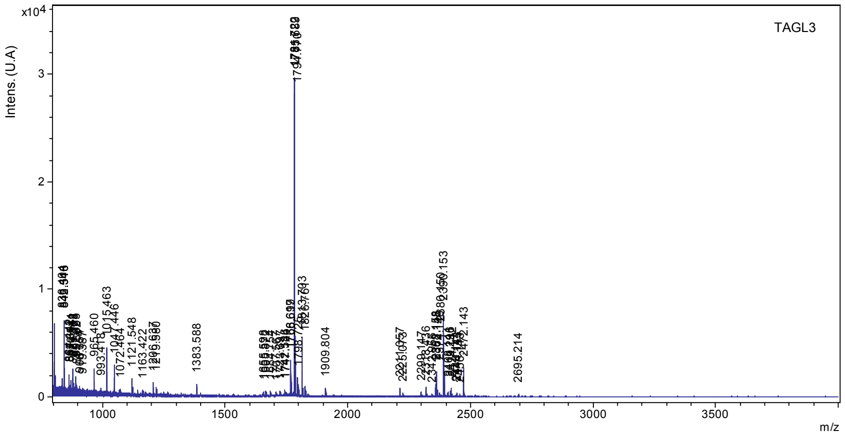
TAGL3
Intens. (U.A)
x10^4
m/z
1383.588
1909.804
2695.214
2472.143

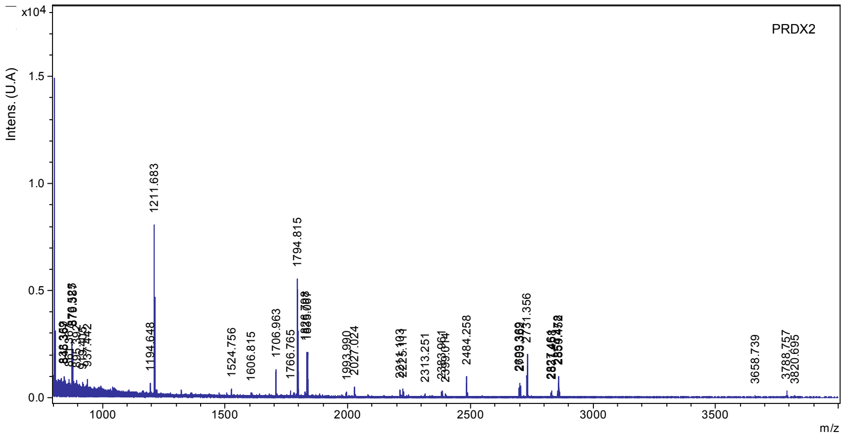
PRDX2
Intens. (U.A)
x10^4
m/z
1211.683
1794.815
1194.648
1524.756
1606.815
1706.963
1766.765
2484.258
3658.739
3820.695

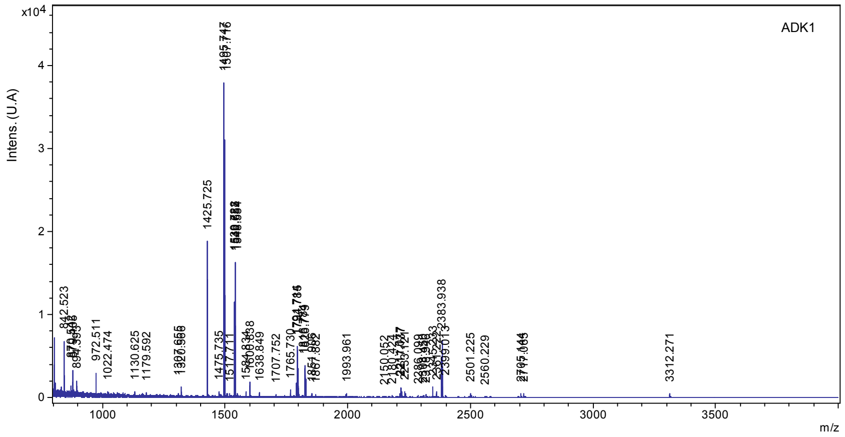
ADK1
Intens. (U.A)
x10^4
m/z
1425.725
842.523
972.511
1022.474
1130.625
1179.592
2383.938
2501.225
2560.229
3312.271

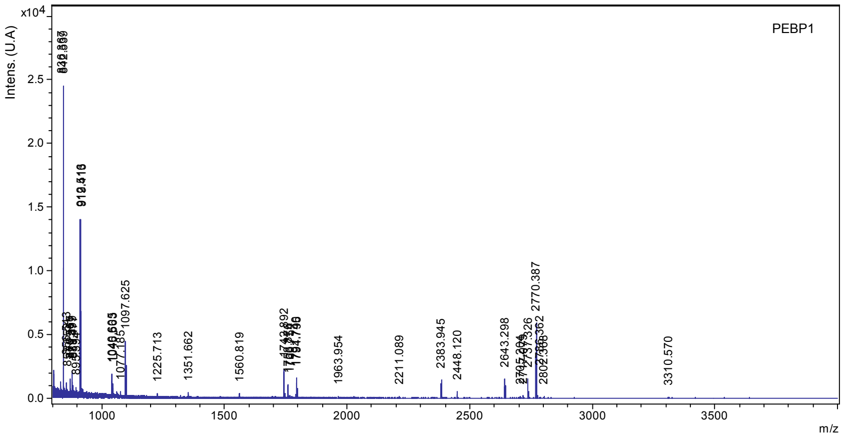
PEBP1
x10^4
Intens. (U.A)
m/z
1097.625
1225.713
1351.662
1560.819
1963.954
2211.089
2383.945
2448.120
2643.298
2770.387
3310.570

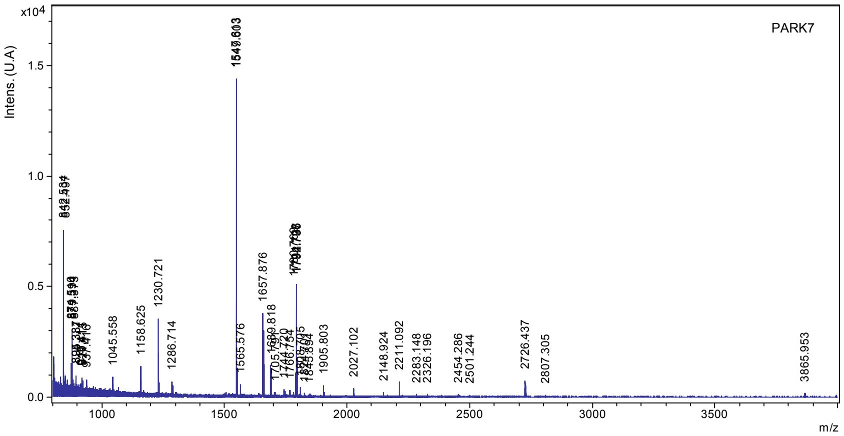
PARK7
x10^4
Intens. (U.A)
m/z
1045.558
1158.625
1230.721
1286.714
1565.576
1657.876
1905.803
2027.102
2148.924
2211.092
2283.148
2326.196
2454.286
2501.244
2726.437
2807.305
3865.953

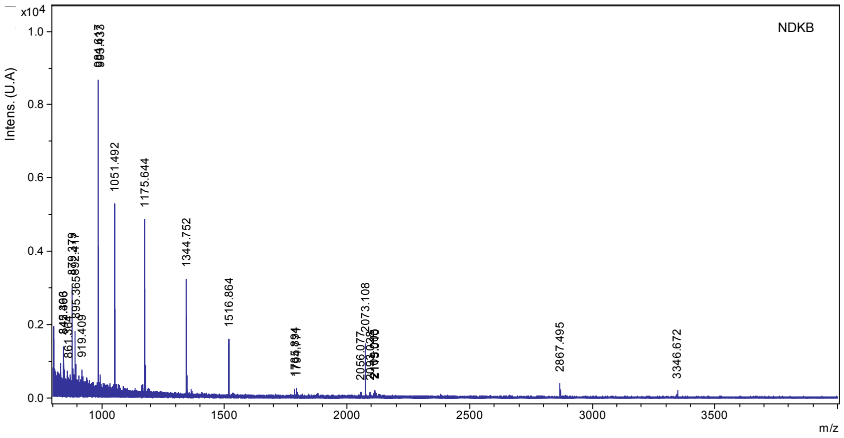
NDKB
x10^4
Intens. (U.A)
m/z
919.409
1051.492
1175.644
1344.752
1516.864
2073.108
2867.495
3346.672

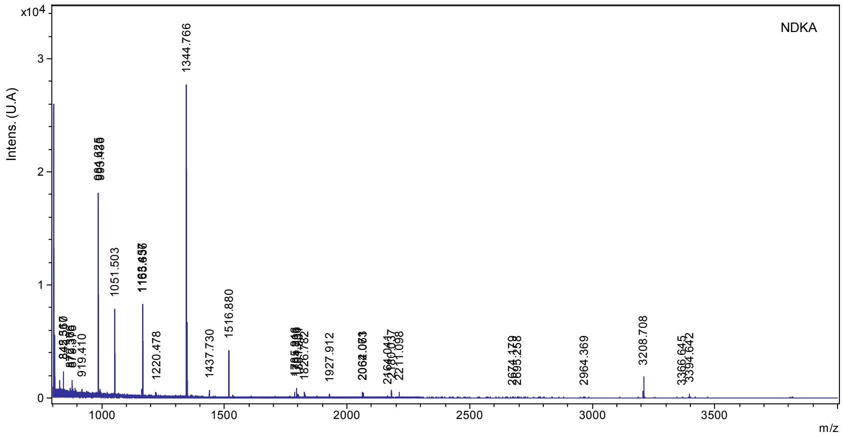
NDKA
x10^4
Intens. (U.A)
m/z
919.410
1051.503
1220.478
1344.766
1437.730
1516.880
1826.782
1927.912
2211.098
2964.369
3208.708

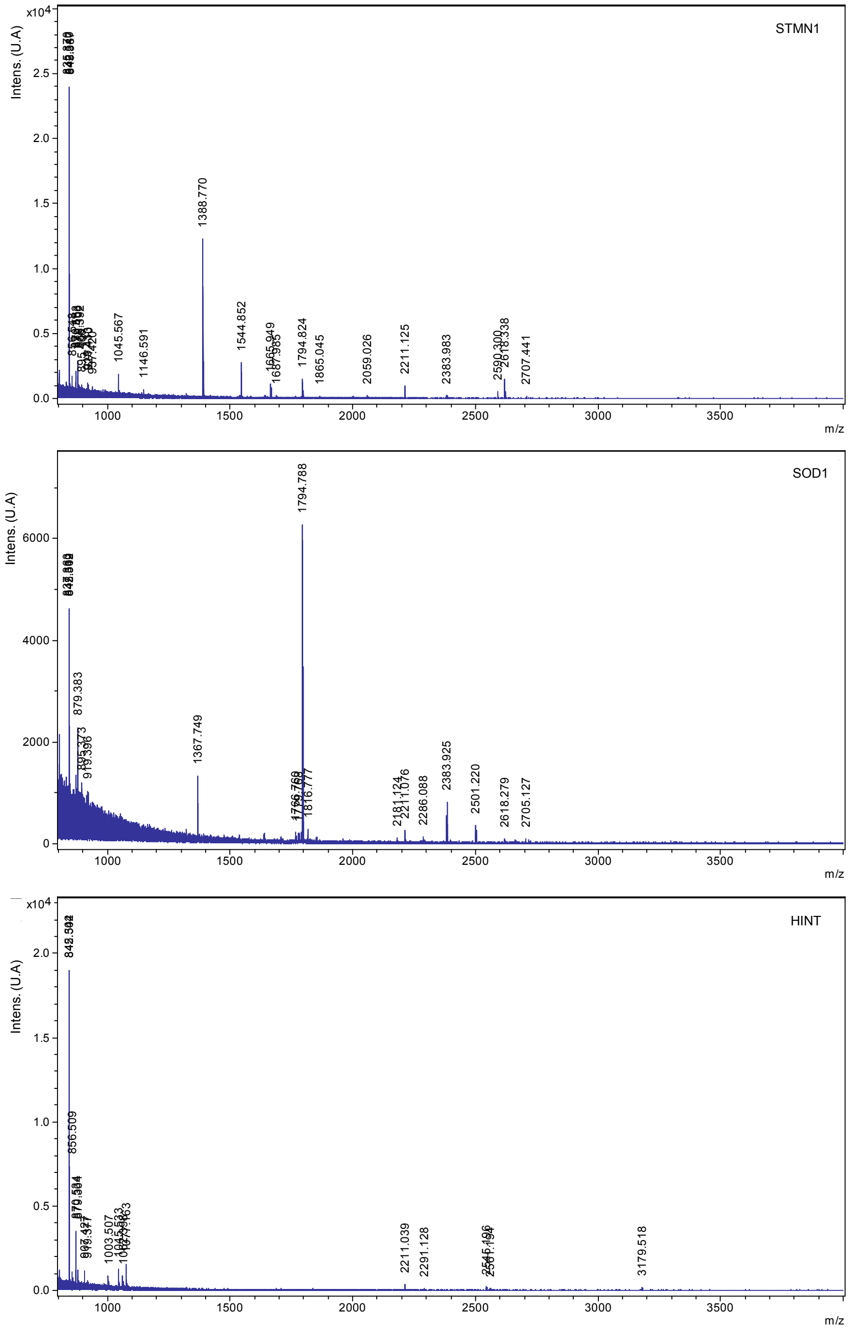
STMN1
Intens. (U.A)
x10^4
1388.770
1544.852
1045.567
1146.591
1794.824
1865.045
2059.026
2211.125
2383.983
2618.338
2707.441
m/z
SOD1
Intens. (U.A)
1794.788
879.383
919.396
1367.749
1816.777
2211.076
2286.088
2383.925
2501.220
2618.279
2705.127
m/z
HINT
Intens. (U.A)
x10^4
856.509
1003.507
2211.039
2291.128
3179.518
m/z

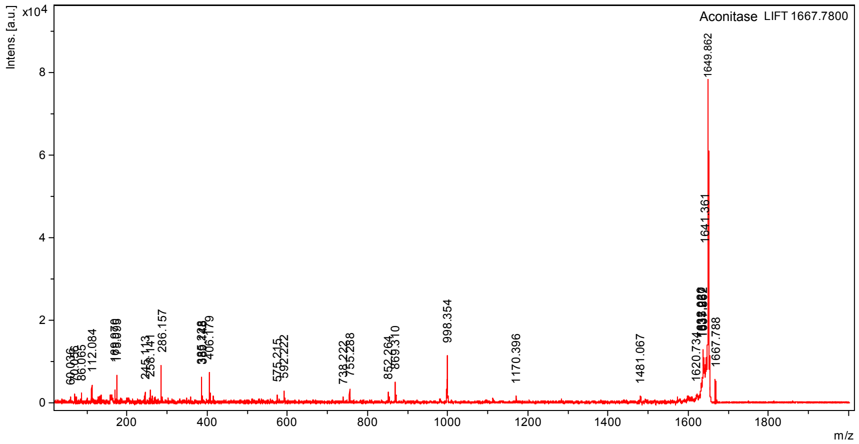
Aconitase LIFT 1667.7800
Intens. [a.u.]
x10^4
m/z
1649.862
998.354

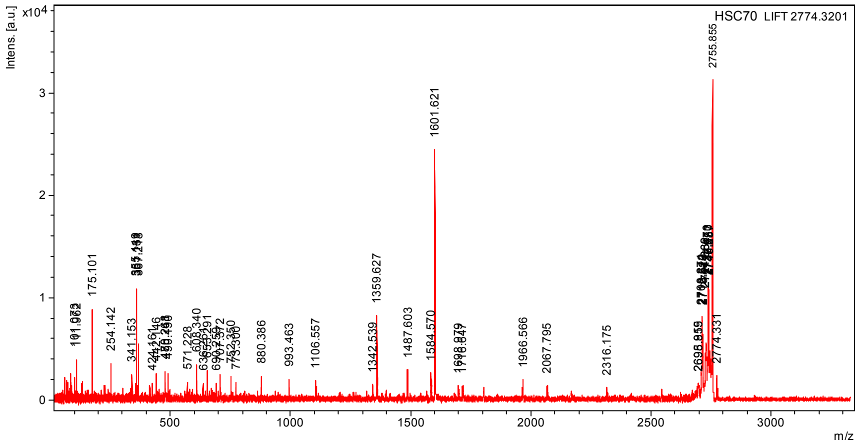
HSC70 LIFT 2774.3201
Intens. [a.u.]
x10^4
m/z
2755.855
1601.621

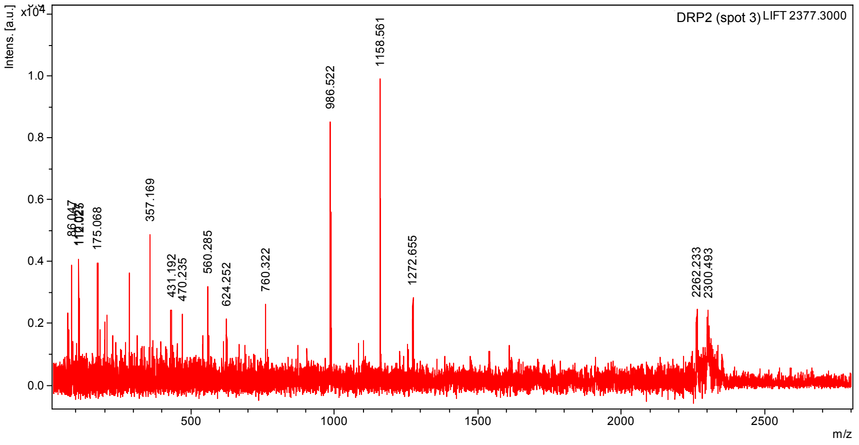
DRP2 (spot 3) LIFT 2377.3000
Intens. [a.u.]
x10^4
m/z
1158.561
986.522

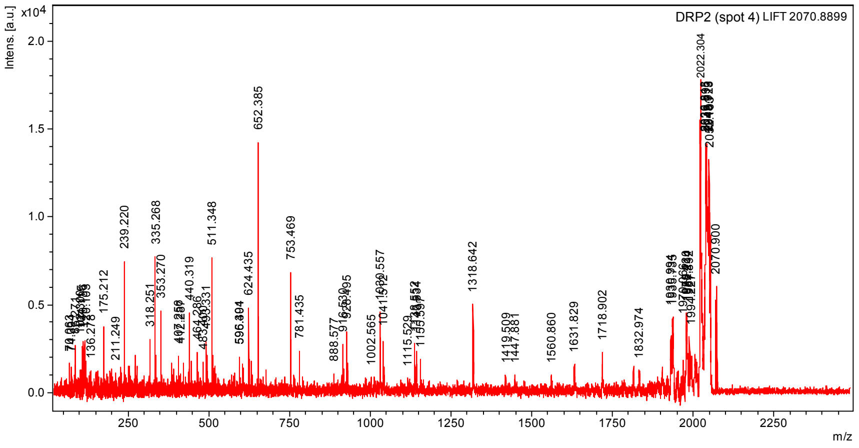
DRP2 (spot 4) LIFT 2070.8899
Intens. [a.u.]
x10^4
m/z
2022.304
652.385

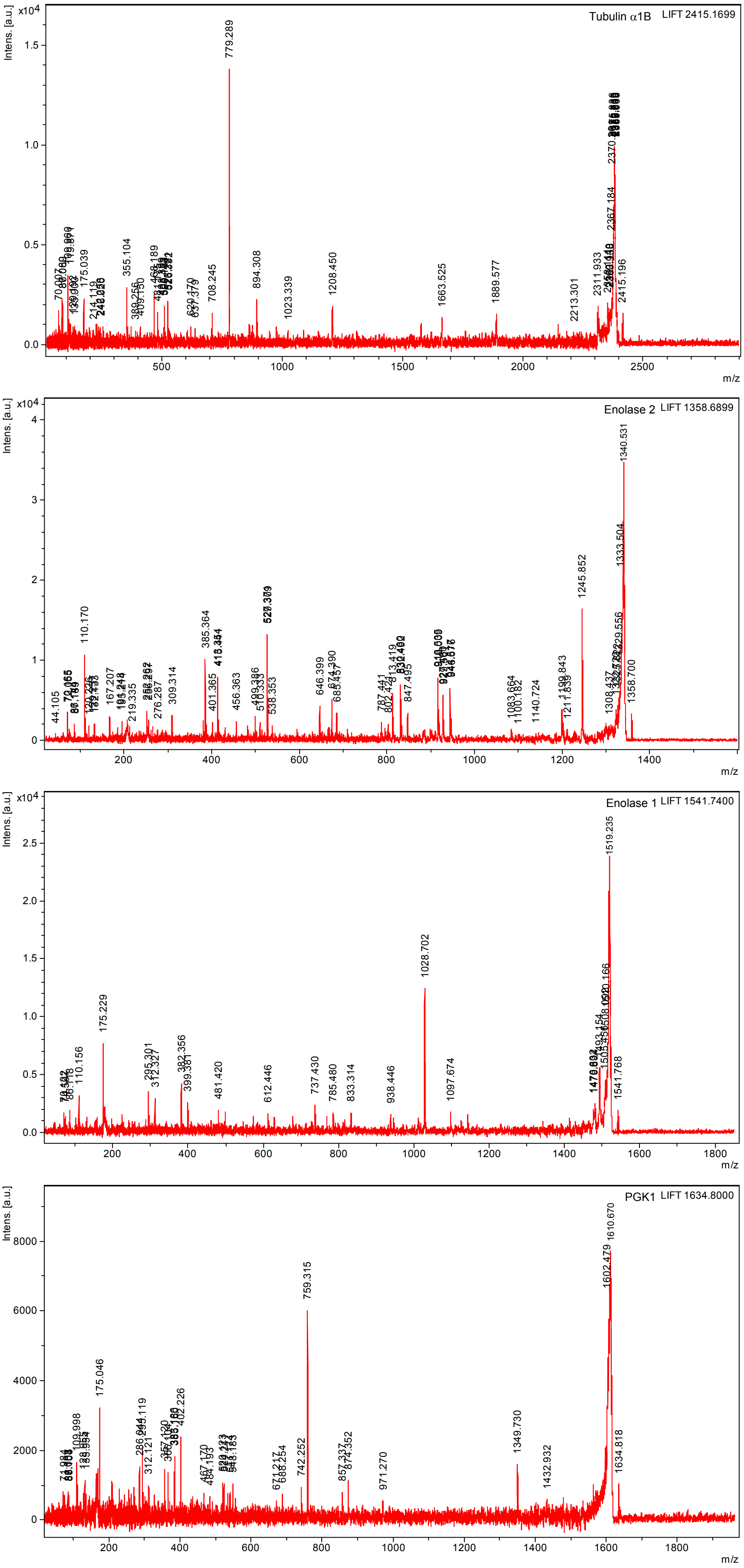
Tubulin α1B LIFT 2415.1699
Enolase 2 LIFT 1358.6899
Enolase 1 LIFT 1541.7400
PGK1 LIFT 1634.8000
Intens. [a.u.]
m/z

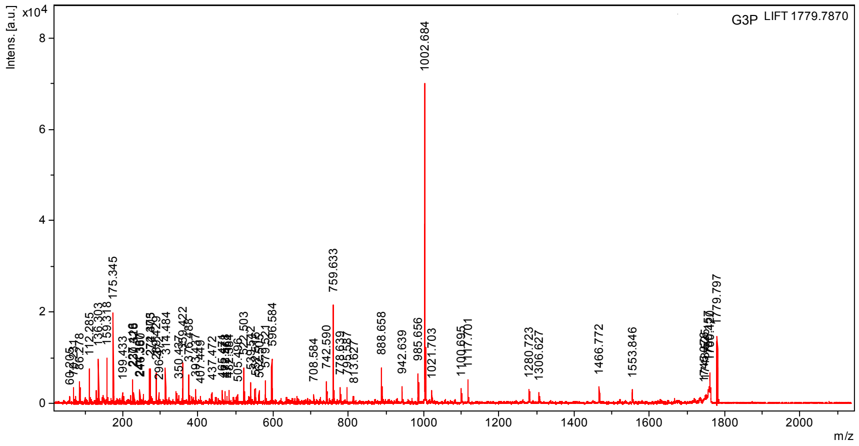
G3P
LIFT 1779.7870
Intens. [a.u.]
x10^4
m/z
1002.684
759.633
175.345
1779.797
888.658
1466.772
1553.846
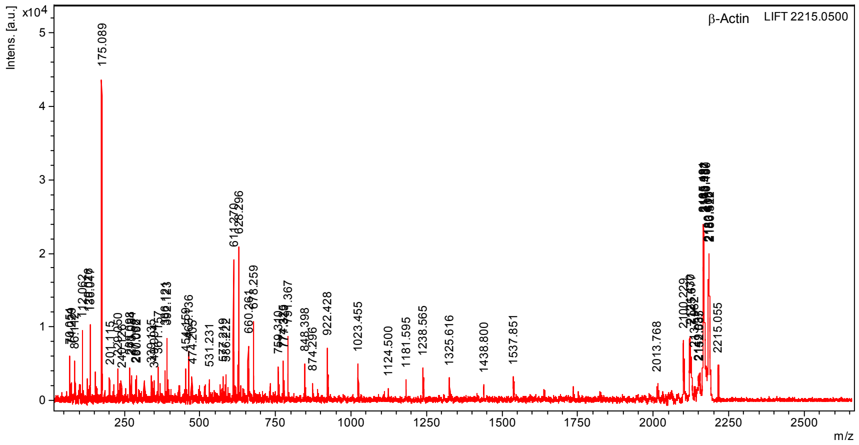
β-Actin
LIFT 2215.0500
Intens. [a.u.]
x10^4
m/z
175.089
628.296
678.259
922.428
1023.455
1238.565
1325.616
1438.800
1537.851
2013.768
2215.055
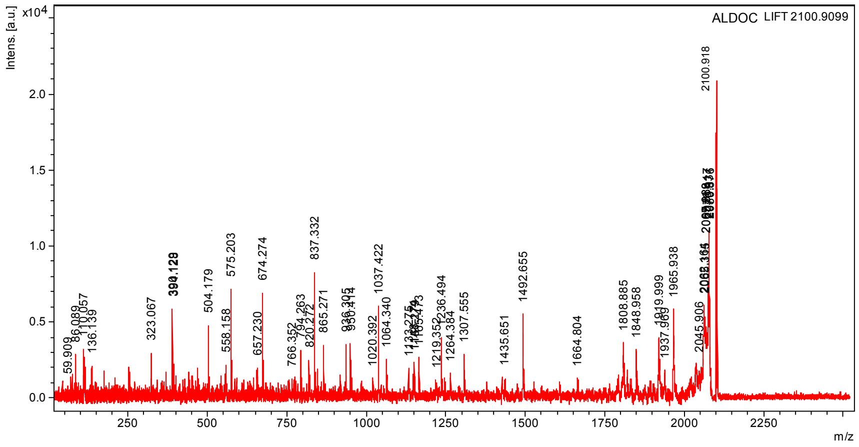
ALDOC
LIFT 2100.9099
Intens. [a.u.]
x10^4
m/z
2100.918
837.332
674.274
575.203
1037.422
1492.655
1965.938
1808.885
1664.804
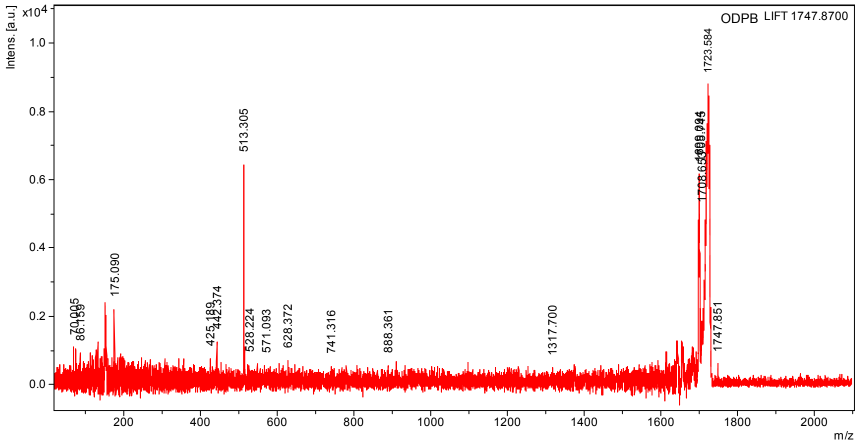
ODPB
LIFT 1747.8700
Intens. [a.u.]
x10^4
m/z
1723.584
513.305
175.090
1317.700
1747.851
888.361
741.316

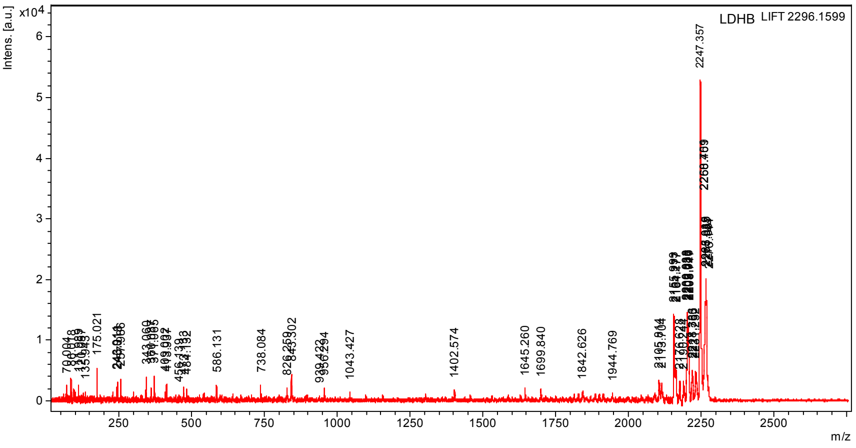
LDHB LIFT 2296.1599
Intens. [a.u.]
x10^4
m/z
2247.357
2260.469
1402.574
1645.260
1699.840
1842.626
1944.769
1043.427
586.131
738.084
175.021

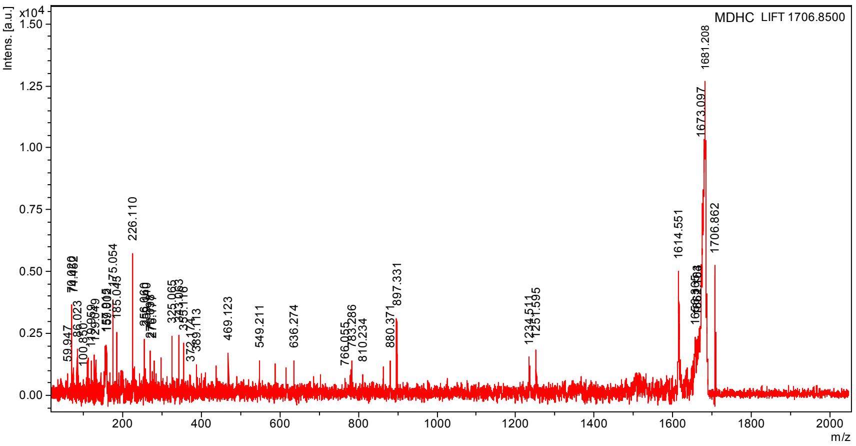
MDHC LIFT 1706.8500
Intens. [a.u.]
x10^4
m/z
1681.208
1673.097
1614.551
1706.862
226.110
175.054
897.331
880.371
469.123
549.211
636.274
1234.511
1251.535

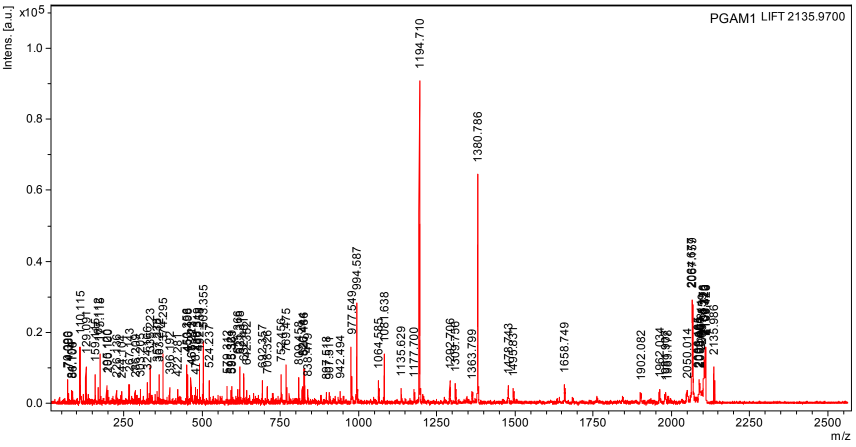
PGAM1 LIFT 2135.9700
Intens. [a.u.]
x10^5
m/z
1194.710
1380.786
994.587
1081.638
1658.749
1902.082
2135.986

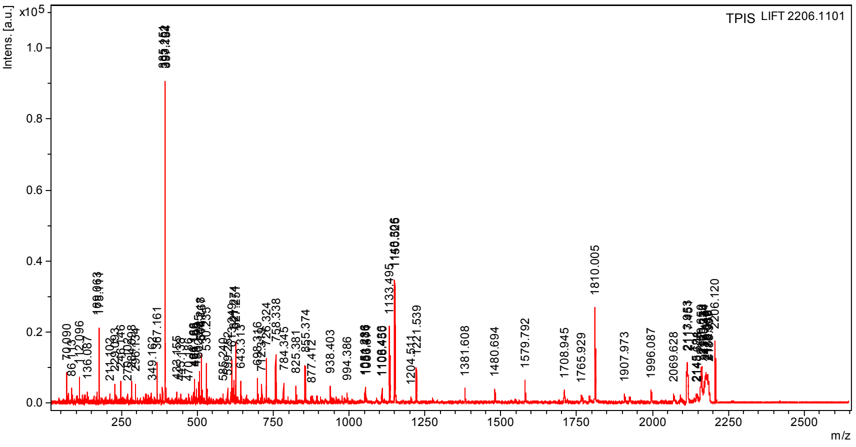
TPIS LIFT 2206.1101
Intens. [a.u.]
x10^5
m/z
1810.005
2206.120
367.161
1221.539
1381.608
1480.694
1579.792
1708.945
1765.929
1907.973
1996.087
2069.628

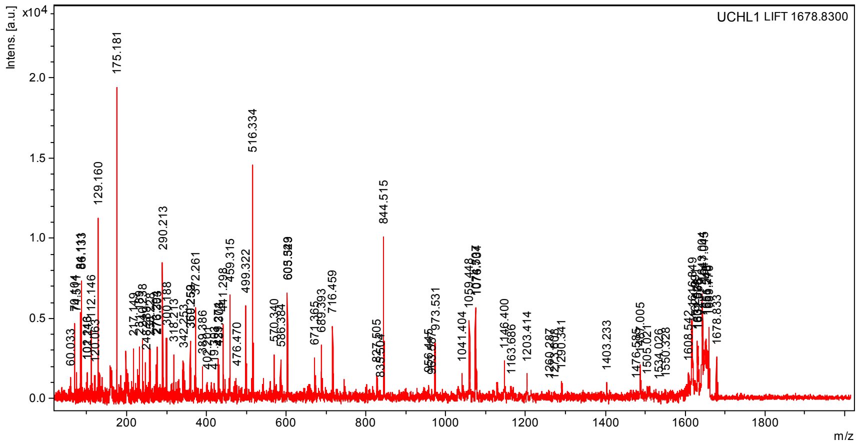
UCHL1 LIFT 1678.8300
Intens. [a.u.]
x10^4
m/z

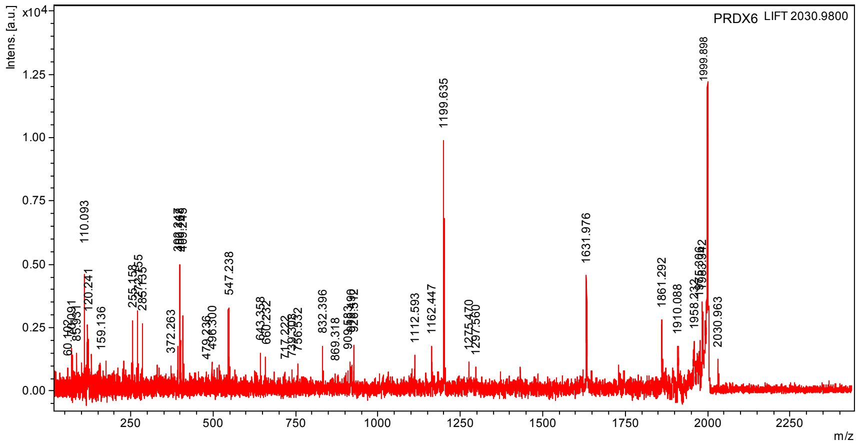
PRDX6 LIFT 2030.9800
Intens. [a.u.]
x10^4
m/z

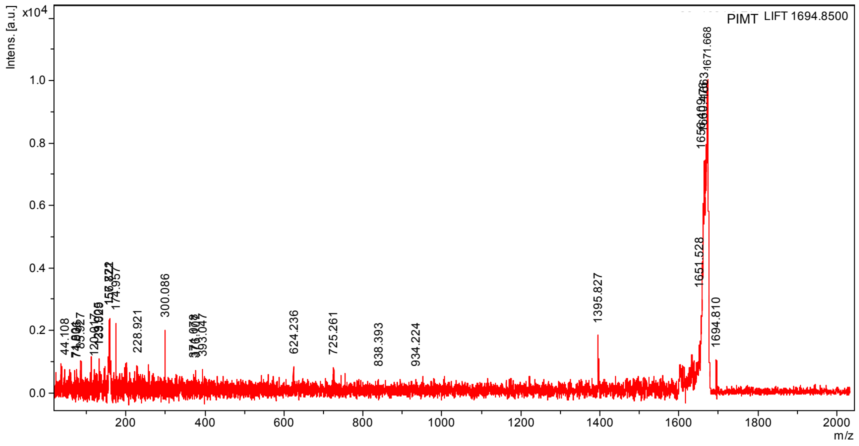
PIMT LIFT 1694.8500
Intens. [a.u.]
x10^4
m/z

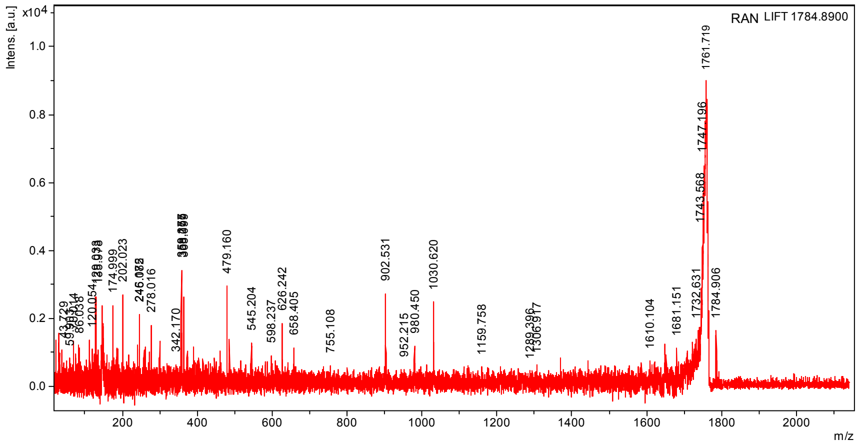
RAN LIFT 1784.8900
Intens. [a.u.]
x10^4
m/z

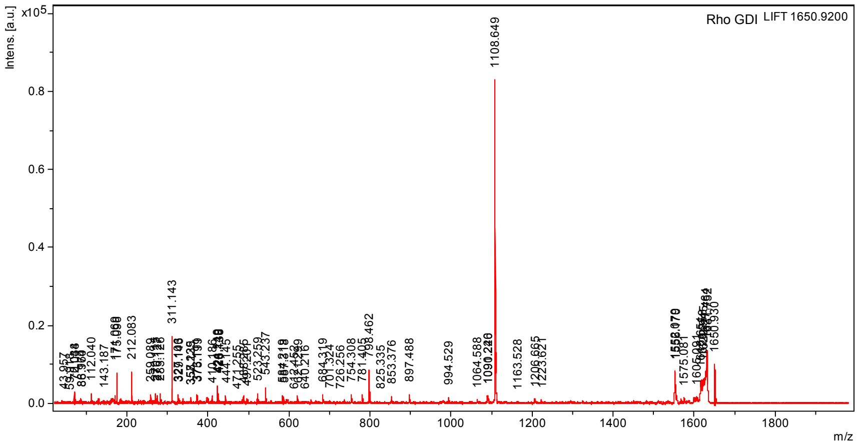
Rho GDI LIFT 1650.9200
Intens. [a.u.]
x10^5
m/z
1108.649
311.143
212.083
798.462
1650.930

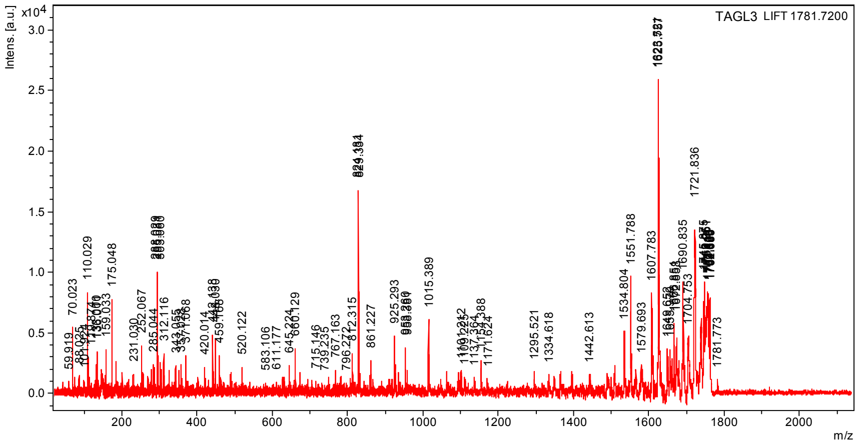
TAGL3 LIFT 1781.7200
Intens. [a.u.]
x10^4
m/z
1721.836
1551.788
1607.783
1690.835
1015.389
110.029
175.048
1781.773

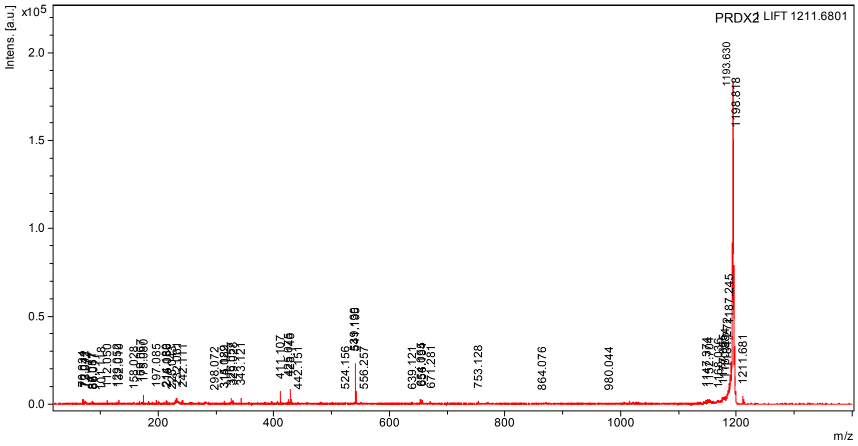
PRDX2 LIFT 1211.6801
Intens. [a.u.]
x10^5
m/z
1193.630
1198.818
1211.681
980.044
864.076
753.128

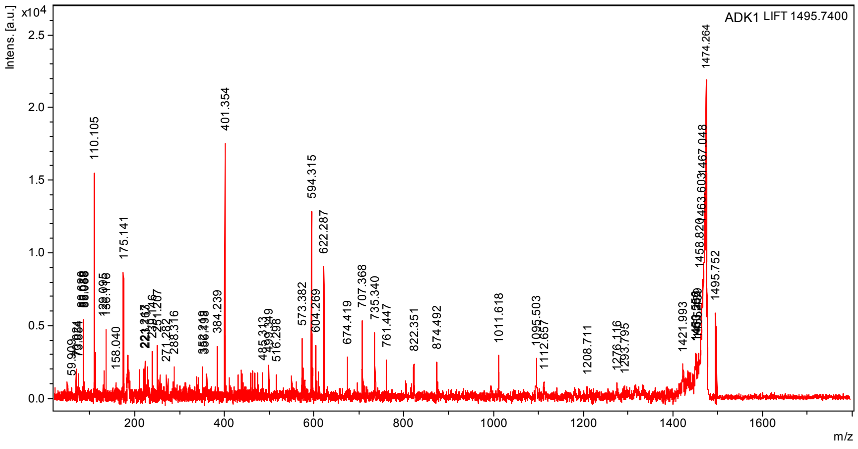
ADK1 LIFT 1495.7400
Intens. [a.u.]
x10^4
m/z
1474.264
401.354
110.105
594.315
622.287
175.141
1495.752
1011.618

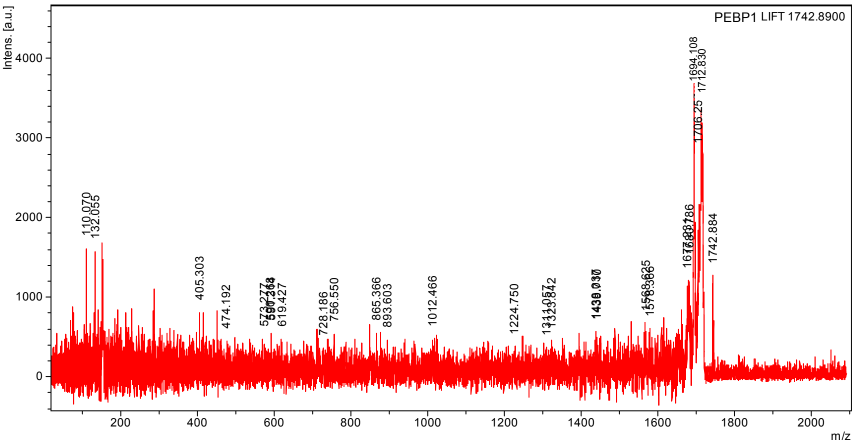
PEBP1 LIFT 1742.8900
Intens. [a.u.]
m/z
1694.108
1712.830
1742.884
110.070
132.055
405.303
474.192
1012.466
1224.750

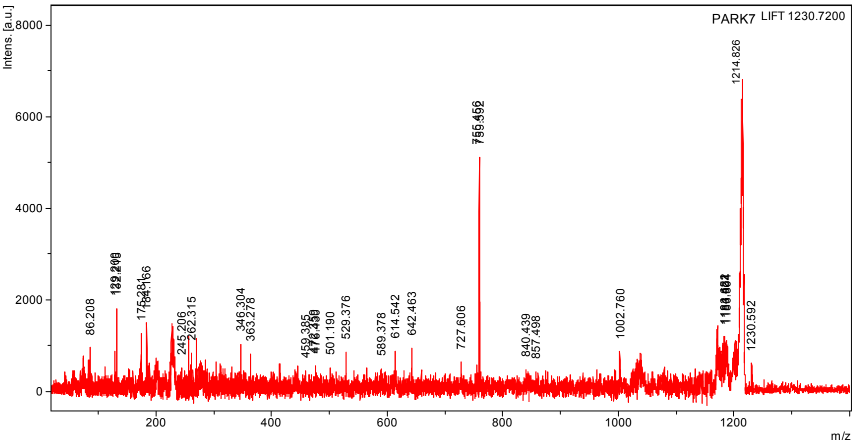
PARK7 LIFT 1230.7200
Intens. [a.u.]
m/z
1214.826
1230.592
1002.760
727.606
642.463
614.542
589.378
529.376
501.190
86.208

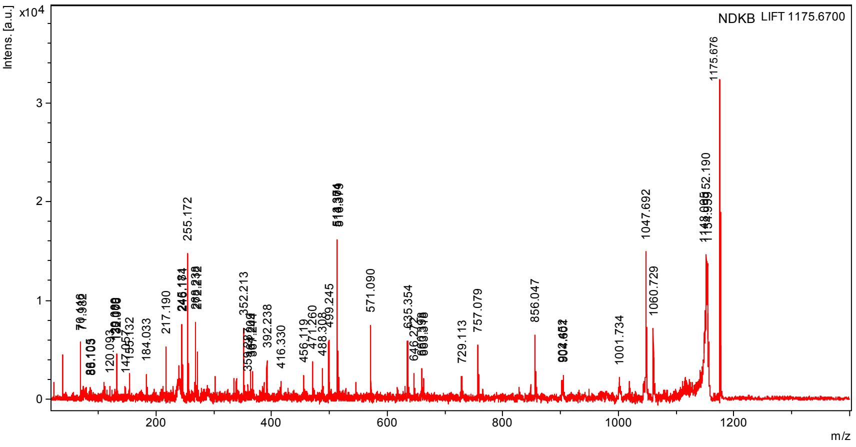
NDKB LIFT 1175.6700
Intens. [a.u.]
x10^4
m/z
1175.676
1047.692
1060.729
1001.734
856.047
757.079
729.113
571.090
255.172
217.190
184.033

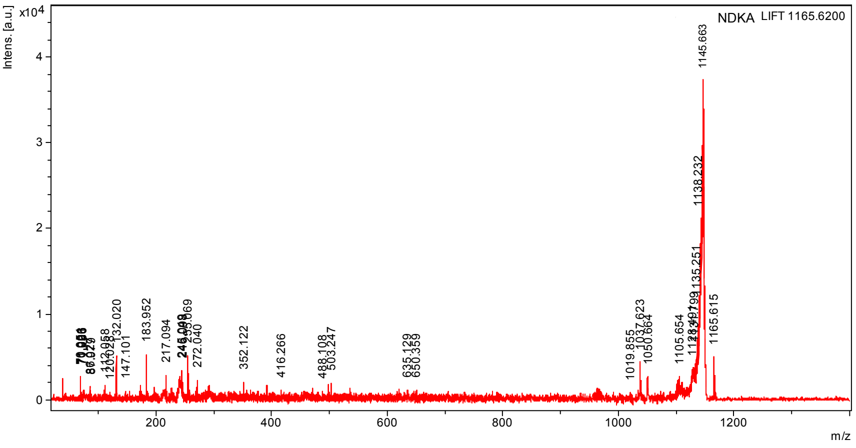
NDKA LIFT 1165.6200
Intens. [a.u.]
x10^4
m/z
1145.663
1138.232
1165.615
1105.654
183.952
147.101
217.094
272.040
352.122
416.266

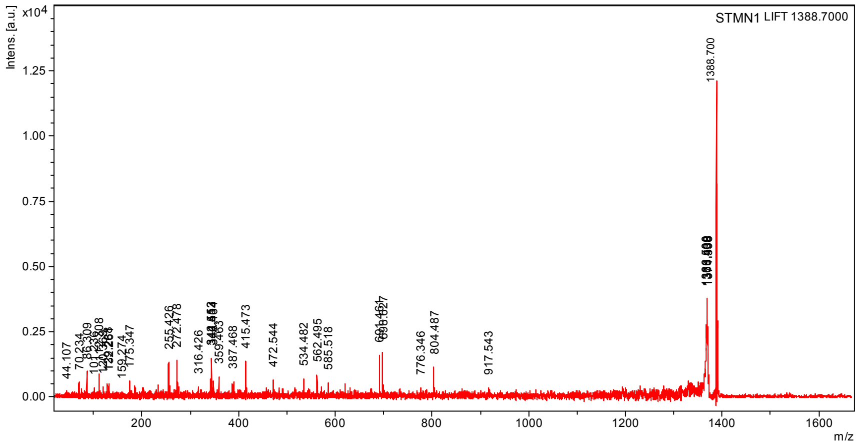
STMN1 LIFT 1388.7000
Intens. [a.u.]
m/z

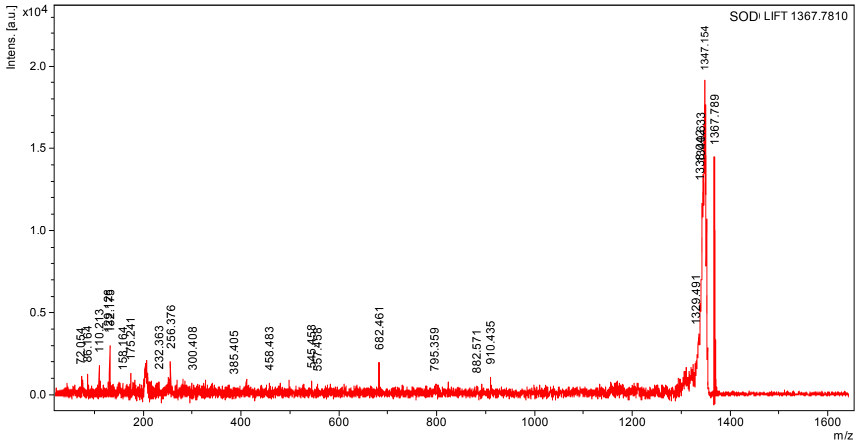
SOD LIFT 1367.7810
Intens. [a.u.]
m/z

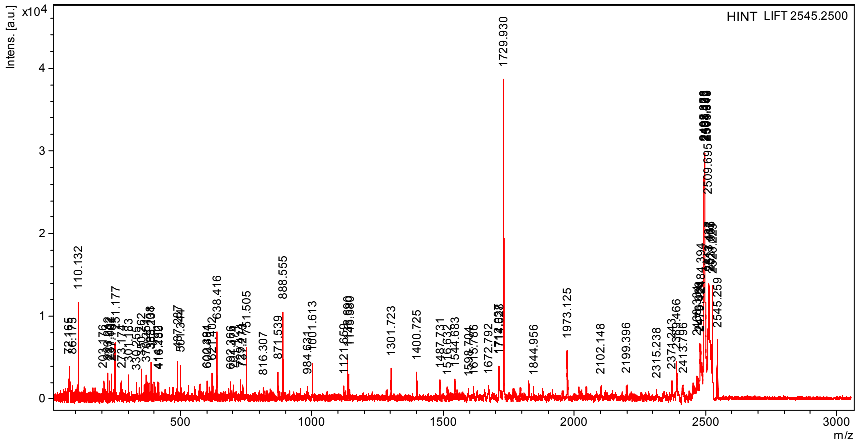
HINT LIFT 2545.2500
Intens. [a.u.]
m/z
